# Distributed Authorization in IoT and Edge Cybersecurity: A Standards-Informed Analytical Framework and Taxonomy

**DOI:** 10.3390/s26206522

**Published:** 2026-10-14

**Authors:** Ryu Watanabe, Toshiaki Tanaka, Jun Kurihara, Toru Nakamura, Ayumu Kubota, Kouichi Sakurai

**Affiliations:** 1 KDDI Research, Inc., 2-1-15 Ohara, Fujimino 356-8502, Saitama, Japan; 2 Graduate School and Faculty of Information Science and Electrical Engineering, Kyushu University, 744 Motooka, Nishi-ku, Fukuoka 819-0395, Fukuoka, Japan; 3 Graduate School of Information Science, University of Hyogo, 7-1-28 Minatojima-minamimachi, Chuo-ku, Kobe 650-0047, Hyogo, Japan; 4 Department of Computer Science, School of Computing, Institute of Science Tokyo, 2-12-1 Ookayama, Meguro-ku, Tokyo 152-8552, Japan; 5 Department of Computer Science, Faculty of Information Technology, Setagaya Campus, Tokyo City University, 1-28-1 Tamazutsumi, Setagaya-ku, Tokyo 158-8557, Japan

**Keywords:** distributed authorization, access control, zero trust architecture, IoT security, edge computing, distributed ledger technology, sensor networks

## Abstract

Authorization is a core function of access control in distributed systems, where decisions and enforcement may span heterogeneous components and administrative domains. This paper presents a standards-informed taxonomy and applies it in a systematic mapping study of 90 studies on distributed authorization in IoT, edge/fog, sensor/WSN, and vehicular/V2X environments, available from 2020 through June 2026. The studies are classified according to six dimensions: policy governance, decision basis, identity and attribute management, PDP–PEP architecture, trust establishment, and operational assurance and auditability. We also examine their correspondence with authorization-relevant Zero Trust Architecture tenets and analyze the functional roles of distributed ledger technologies and smart contracts. The mapping shows that Distributed PDP/Local PEP is the dominant architectural pattern, while architectural distribution does not necessarily imply decentralized policy governance, identity and attribute management, or trust establishment. Within the mapped corpus, DLTs and/or smart contracts are widely used, but their use alone does not establish decentralization or complete authorization-lifecycle control. Overall, the results support treating distributed authorization as a combination of related but distinct design dimensions and highlight remaining challenges in lifecycle control, interoperability, delegation-oriented authorization, and operational evaluation.

## 1. Introduction

Authorization and access control are fundamental concepts in information security. Numerous standards, technical studies, and guidelines address authorization and access control, including policy-based authorization frameworks such as eXtensible Access Control Markup Language (XACML) [1,2], token-based authorization frameworks such as OAuth 2.0 [3], and identity-federation mechanisms such as OpenID Connect (OIDC) [4]. Recent OIDC profiles have also been proposed for self-sovereign identity (SSI) environments [5]. However, terminology and conceptual scope remain inconsistent across studies. The same mechanism may be described using different terms, while the same term may refer to different architectural or functional assumptions. These inconsistencies make it difficult to compare authorization models systematically.

Classical access control models remain important reference points [6,7]. However, advances in Information and Communication Technology (ICT) have introduced environments and requirements that were not fully considered in classical models. Examples include cloud computing, which enables shared computing resources [8,9]; edge computing, which places computation and services closer to data sources [9,10]; the Internet of Things (IoT), which involves large numbers of heterogeneous and resource-constrained devices [10]; and SSI, which enables users or organizations to hold and present their own identity information and credentials, often with support from distributed ledger technologies (DLTs) [11,12]. These developments have motivated numerous authorization and access-control mechanisms designed for distributed and heterogeneous environments.

These challenges are particularly visible in IoT, edge/fog, sensor/WSN, and vehicular/V2X cybersecurity environments. In these settings, devices, gateways, edge servers, cloud services, vehicles, roadside infrastructure, and cross-domain applications may interact under heterogeneous administrative and trust assumptions. Authorization is therefore not necessarily performed by a single centralized component. Policy governance, decision making, enforcement, identity and attribute management, trust establishment, and authorization-lifecycle control may instead be distributed across multiple components or administrative domains.

Given the number and diversity of proposed models, it remains difficult to obtain a consistent view of distributed authorization. This paper focuses specifically on authorization because it determines whether a requested operation should be permitted, based on policies, attributes, roles, relationships, rules, or capabilities. We focus on distributed environments because authorization-relevant functions may span multiple systems, authorities, and organizational domains, even when some components remain logically centralized.

In this study, the term *distributed* broadly denotes an architecture in which at least one authorization-relevant function—such as policy governance, decision logic, identity or attribute management, trust establishment, or enforcement—is explicitly divided across multiple components or administrative domains. By contrast, we use *decentralized* more narrowly to denote reduced dependence on a single controlling authority or trust root. A system may therefore be distributed in architectural terms while retaining centralized policy authority, identity management, or trust establishment. Distinguishing these concepts is essential for analyzing how authorization responsibilities are actually allocated.

Authentication, authorization, and audit are treated as the principal functional components of access control. Authentication establishes or verifies the identity and relevant characteristics of a subject. Authorization evaluates whether a requested action should be permitted. Audit records authorization-related actions and state changes for monitoring, accountability, and operational assurance. The present study focuses primarily on authorization, while treating authentication-related identity and attribute information, trust establishment, and auditability as supporting dimensions that influence authorization design.

To analyze these dimensions consistently, we organize the literature using criteria derived from historical access control models and modern security standards. The framework examines policy governance, decision basis, identity and attribute management, Policy Decision Point (PDP)–Policy Enforcement Point (PEP) architectural placement, trust establishment, and operational assurance. Selected Zero Trust Architecture (ZTA) tenets are additionally used as supplementary analytical dimensions for examining session scope, dynamic policy inputs, and authorization-lifecycle enforcement.

Prior surveys have examined access control and authorization from several perspectives. These include cloud-oriented distributed authentication and authorization models and requirements [13], general definitions and model taxonomies [14], cross-domain identity and access management issues and solution spaces [15], security- and privacy-focused reviews of IoT smart-home access control [16], blockchain-assisted access control with a networking focus [17], broader blockchain-centered reviews of access control models [18], and authorization models for IoT environments [19]. Table 1 compares their analytical coverage with that of the present study.

The comparison shows that prior reviews provide important domain-specific, model-oriented, identity-management, or blockchain-centered perspectives, but do not jointly operationalize policy governance, decision basis, identity and attribute management, PDP–PEP placement, trust establishment, and operational assurance as separate analytical dimensions. They also do not combine these dimensions with authorization-oriented ZTA indicators and explicit functional coding of DLT/SC roles. The present study therefore complements rather than replaces these earlier reviews by providing a common analytical basis for comparing distributed authorization designs across heterogeneous application environments.

This distinction is important because distributing PDPs, policy stores, or enforcement components does not necessarily decentralize policy authority, identity management, or trust roots. Similarly, the use of DLTs or smart contracts (SCs) may support policy evaluation, shared state, trust anchoring, coordination, or auditability without guaranteeing dynamic reauthorization or revocation. By separating architectural distribution from governance, identity management, trust establishment, and lifecycle behavior, the proposed framework enables comparison of design choices that are often combined or treated implicitly in earlier surveys.

The empirical mapping is restricted to studies available from 2020 through 30 June 2026. The year 2020 is used as a practical temporal scoping boundary rather than as an assertion that distributed authorization originated in that year. NIST SP 800-207 was finalized in 2020 and established a resource-centric Zero Trust Architecture baseline that is also used in this study as a supplementary analytical reference [20]. Accordingly, 2020 provides a reproducible calendar-year boundary for focusing the empirical mapping on contemporary distributed authorization designs associated with this period of modern zero-trust and distributed-system development. Earlier authorization and access-control research remains important to the conceptual foundations of the field and is therefore incorporated in Section 2 and Section 3, but is not counted as part of the empirical mapping corpus.

Against this background, this paper introduces a standards-informed analytical framework and taxonomy for characterizing authorization in distributed environments. Using this framework, we synthesize recent proposals under a consistent analytical baseline and identify recurring architectural patterns, governance assumptions, trust mechanisms, and authorization-lifecycle characteristics. To the best of our knowledge, few prior studies jointly analyze distributed authorization models from the perspectives of policy governance, decision basis, identity and attribute management, PDP–PEP placement, trust establishment, and operational assurance, while also relating the selected models to authorization-relevant ZTA tenets.

This systematic mapping study is guided by the following research questions:

**RQ1:** Which dimensions and labels can be used to systematically map distributed authorization studies in terms of policy governance, decision basis, identity and attribute management, architectural placement, trust establishment, and operational assurance and auditability?
**RQ2:** What recurring architectural patterns and design characteristics are observed within the selected corpus of recent distributed authorization studies?
**RQ3:** How do the selected studies correspond to authorization-relevant aspects of ZTA tenets T3, T4, and T6 concerning session scope, dynamic policy inputs, and authorization-lifecycle enforcement?

The main contributions of this paper are as follows:

1. We develop a standards-informed analytical framework and coding scheme (C1–C6) for systematically mapping distributed authorization studies across policy governance, decision basis, identity and attribute management, PDP–PEP architectural placement, trust establishment, and operational assurance and auditability.
2. We apply the framework to a selected corpus of 90 studies from IoT, edge/fog, sensor/WSN, and vehicular/V2X environments and map recurring architectural patterns, governance structures, decision models, identity and attribute management approaches, trust assumptions, and authorization-lifecycle characteristics.
3. We examine the correspondence of the selected studies with authorization-relevant aspects of ZTA tenets T3, T4, and T6 and analyze the functional roles of DLTs and SCs in the mapped authorization designs.

The remainder of this paper is organized as follows. Section 2 summarizes reference concepts and architectures derived from security standards and used as the analytical baseline. Section 3 provides a historical overview of access control models. Section 4 presents the classification framework, systematic mapping method, mapping results, synthesis, and future research directions. Finally, Section 5 concludes the paper.

## 2. Reference Concepts and Architectures from Security Standards

This section summarizes the standards-derived concepts used to motivate the classification criteria, focusing on authorization functions, the reference monitor, and the logical placement of Policy Decision Point (PDP) and Policy Enforcement Point (PEP) components. For readability, the main abbreviations used throughout this study are listed in the Abbreviations section.

### 2.1. Authentication, Authorization, and Audit

Authentication verifies the identity and relevant characteristics of an entity, authorization determines whether a requested operation should be permitted, and audit records access-related events and state changes for accountability and operational assurance. These functions are reflected in major security references, including the Trusted Computer System Evaluation Criteria (TCSEC) [21], the Open Systems Interconnection security architecture [22], NIST SP 800-12 [23], and ISO/IEC 27001 [24]. In this study, authentication and audit are considered only insofar as identity and attribute information, trust establishment, auditability, and change control affect authorization decisions, enforcement, or lifecycle management.

### 2.2. Reference Monitor as a Centralized Enforcement Baseline

The reference monitor provides a conceptual baseline for centrally mediated access control through complete mediation, protection from unauthorized modification, and analyzable enforcement [21,25,26,27]. In this study, it serves as a baseline for interpreting how authorization decision and enforcement functions are separated and placed in centralized, distributed, hierarchical, and resource-side PDP–PEP configurations.

### 2.3. ZTA and Logical Components for Access Control

Zero Trust Architecture (ZTA) provides a modern reference architecture for enterprise access control that emphasizes least privilege, contextual policy evaluation, and continued verification [20]. In this study, selected ZTA tenets are used only as supplementary analytical dimensions for session scope, dynamic policy inputs, and authorization-lifecycle mechanisms, rather than as universal requirements or measures of overall ZTA compliance. Table 2 summarizes the seven ZTA tenets (Each explanation is a summary by the authors. Please refer to the original [20] for the exact descriptions) while Table 3 and Table 4 summarize the logical components and information sources most relevant to authorization analysis. Within ZTA, the PDP comprises the Policy Engine (PE) and Policy Administrator (PA), while the PEP controls access to the protected resource; this logical separation provides a baseline for the C4 architectural classification. Related IETF NMOP work on distributed authorization-policy sharing complements this perspective by addressing policy fragmentation, validation, versioning, and cross-domain exchange [28].

## 3. Historical Evolution of Access Control Models

This section summarizes the access-control concepts needed to interpret the mapping criteria, from classical models to RBAC, ABAC, ReBAC, CAP-BAC, UCON, modern authorization specifications, and SSI.

### 3.1. Classical Models of Access Control

Classical models such as Bell–LaPadula [29], Biba [30], Clark–Wilson [31], Chinese Wall [32], LOMAC [33], and DTE [34] provide historical baselines developed largely under centralized policy-governance and enforcement assumptions.

### 3.2. Modern Access Control, Authorization, and Identity Models

#### 3.2.1. Role-Based Access Control (RBAC)

RBAC assigns permissions through roles rather than directly to individual users and was developed from the early Ferraiolo–Kuhn model to the NIST reference model [6,7,35]. In this study, RBAC is treated as a decision-basis category independently of policy governance and architectural placement.

#### 3.2.2. Attribute-Based Access Control (ABAC)

ABAC makes authorization decisions from subject, resource, action, and environmental attributes [36,37] and is broader than RBAC because roles can be represented as one class of subject attribute [38]. Accordingly, RBAC and ABAC are coded as decision bases independently of where policies, attributes, PDPs, and PEPs are managed or deployed. General-purpose policy engines such as the Open Policy Agent (OPA) support practical attribute-aware policy evaluation while leaving administration and enforcement placement to the surrounding architecture [39].

#### 3.2.3. Relationship-Based Access Control (ReBAC)

ReBAC bases authorization on explicitly represented relationships or relationship paths among entities [40]. A study is coded as ReBAC only when such relationships form part of the authorization decision semantics; delegation or ownership information alone is insufficient.

#### 3.2.4. Capability-Based Access Control (CAP-BAC)

A capability represents authority over a protected resource and permitted operations [41] and can be presented to an enforcement component for local validation [42]. This separation of authorization issuance from enforcement is attractive in distributed and resource-constrained environments [43], but capability designs still require explicit treatment of validity, delegation, replay, expiration, and revocation. Because CAP-BAC can use RBAC, ABAC, or rule-based logic to determine issuance, this study records the issuance logic under C2 and CAP-BAC as a C4 architectural subtype rather than as an independent decision basis. Related mechanisms such as NGAC and Macaroons also support distributed policy administration, delegation, or privilege attenuation [44,45].

#### 3.2.5. Usage Control (UCON)

UCON extends access control with authorizations, obligations, conditions, continuity of decision, and attribute mutability [46]. Its continuous-control perspective informs the analysis of session-bounded access, dynamic policy inputs, reauthorization, and revocation under T3, T4, and T6 rather than forming an additional C2 category.

#### 3.2.6. Specifications for Modern Authorization

XACML [1,2] and OAuth 2.0 [3] illustrate policy-based and token-based approaches to modern authorization, respectively.

##### XACML

XACML separates policy administration, information provision, decision making, and enforcement through the PAP, PIP, PDP, and PEP, providing an important reference for the PDP–PEP classification used in this study. Compared with ZTA, XACML represents contextual information explicitly through the PIP, whereas ZTA obtains comparable inputs from multiple identity, asset-state, monitoring, and threat-information sources [20].

##### OAuth 2.0

OAuth 2.0 enables delegated and limited access through authorization grants and access tokens [3] and is commonly combined with OpenID Connect when authentication and identity claims are required. Token-based authorization is not automatically CAP-BAC: CAP-BAC is assigned only when the token or credential represents an independently verifiable authorization grant, while expiration, refresh, revocation, and post-issuance validation are considered separately in the lifecycle analysis. Table 5 summarizes the principal differences between XACML and OAuth 2.0.

##### Federated Identity and OpenID Connect

Traditional enterprise identity management commonly relies on centrally managed directories such as LDAP [48] and Active Directory [49], whereas federation extends identity and authentication across administrative domains through established trust relationships. OpenID Connect (OIDC), an identity layer built on OAuth 2.0 [4], supplies externally issued identity claims that may serve as authorization inputs. Accordingly, federation affects both the placement of identity and attribute management and the trust relationships required to validate cross-domain claims.

#### 3.2.7. Self-Sovereign Identity (SSI)

SSI seeks to give users or organizations greater control over possession and presentation of identity data [50,51]. DIDs and Verifiable Credentials (VCs) provide standardized mechanisms for cryptographically verifiable identifiers and claims [52,53] and can support user-held authorization-relevant information. In this study, SSI primarily affects identity and attribute management and trust establishment; it does not by itself determine the decision basis or PDP–PEP placement.

##### Use of Distributed Ledger Technology in SSI

SSI does not inherently require DLT, although some implementations use ledgers for DIDs, credential schemas, governance information, status data, policy state, or auditability. Examples include smart-contract platforms such as Ethereum and permissioned infrastructures such as Hyperledger Fabric [54,55]. The use of DLT may support trust anchoring, shared state, governance, or auditability, but does not by itself decentralize policy authority or establish complete authorization-lifecycle control.

### 3.3. Historical Overview of Access Control Models

Table 6 summarizes representative historical tendencies using C1–C5; the mappings are illustrative because governance, identity management, trust, and PDP–PEP placement remain implementation-dependent. Classical MAC and DAC commonly assumed a single administrative domain, whereas later models introduced role-, attribute-, relationship-, capability-, and usage-oriented decision and enforcement mechanisms. XACML, OAuth 2.0, OIDC, and SSI further separated policy evaluation, enforcement, token issuance, and identity-claim exchange across systems and domains. This progression motivates the multidimensional taxonomy used below, which maps governance, decision basis, identity and attribute management, PDP–PEP placement, trust establishment, and operational assurance separately rather than assigning a single architectural property to a model or technology.

## 4. Classification of Distributed Authorization Models

This section presents the analytical framework used to systematically map authorization mechanisms in distributed environments. We first define the conceptual scope of distributed authorization and then introduce the classification criteria used to examine the selected studies. The framework separates architectural placement from policy governance, decision basis, identity and attribute management, trust establishment, and operational assurance and auditability.

### 4.1. Foundations for Analyzing Distributed Authorization

As established in Section 2, authorization decisions depend not only on policy semantics but also on identity and attribute information, trust relationships, enforcement placement, and operational feedback. Accordingly, authentication- and audit-related mechanisms are considered in this study only insofar as they provide inputs to, establish trust for, or record and support the lifecycle of authorization decisions.

Distributed authorization cannot be characterized solely by whether multiple PDPs or PEPs are deployed. Authorization-relevant responsibilities may be distributed across policy authorities, identity or attribute providers, decision components, enforcement points, trust anchors, and audit or policy-state infrastructures. Conversely, an architecture may distribute some of these components while retaining centralized control over policy governance, identity management, or trust establishment.

This distinction is particularly important in multi-cloud, cross-organizational, IoT, edge/fog, sensor-network, and vehicular environments. Such settings may require policies and attributes to be interpreted across administrative domains, authorization decisions to incorporate distributed or dynamic information, and enforcement to occur close to protected resources. They may also require mechanisms for credential expiration, revocation propagation, reauthorization, policy updates, and auditable state changes.

The proposed framework therefore analyzes distributed authorization as a combination of separable design dimensions rather than as a single architectural property. This multidimensional perspective provides the basis for the classification criteria introduced in the following subsection.

### 4.2. Analysis of Authorization Models Using Criteria and Dimensions

Building on the conceptual foundation outlined above, this subsection defines the analytical criteria used to systematically map the selected distributed authorization studies. The framework separates six design dimensions: policy governance, decision basis, identity and attribute management, PDP–PEP architectural placement, trust establishment, and operational assurance and auditability.

These dimensions address different questions about an authorization design. Policy governance concerns who controls policy definition and modification; decision basis concerns the information and logic used to determine access; identity and attribute management concerns where authorization-relevant information is issued, maintained, and obtained; PDP–PEP architecture concerns the placement and separation of decision and enforcement functions; trust establishment concerns the mechanisms used to accept identities, attributes, policies, or decisions across domains; and operational assurance and auditability concern the explicitly reported mechanisms for recording, verifying, and controlling authorization-related state changes.

The criteria are used as complementary analytical dimensions rather than as indicators of technical quality, maturity, or effectiveness. Table 7 summarizes the six criteria used in the systematic mapping study.

Table 7 provides traceability from each analytical criterion to the standards and reference concepts that motivate it and summarizes its study-level coding operationalization. These standards and reference concepts inform the analytical dimensions but do not prescribe the study-level labels directly. Complete operational definitions, label boundaries, subtype definitions, and coding rules are provided in Appendix C.

**Figure 1:**
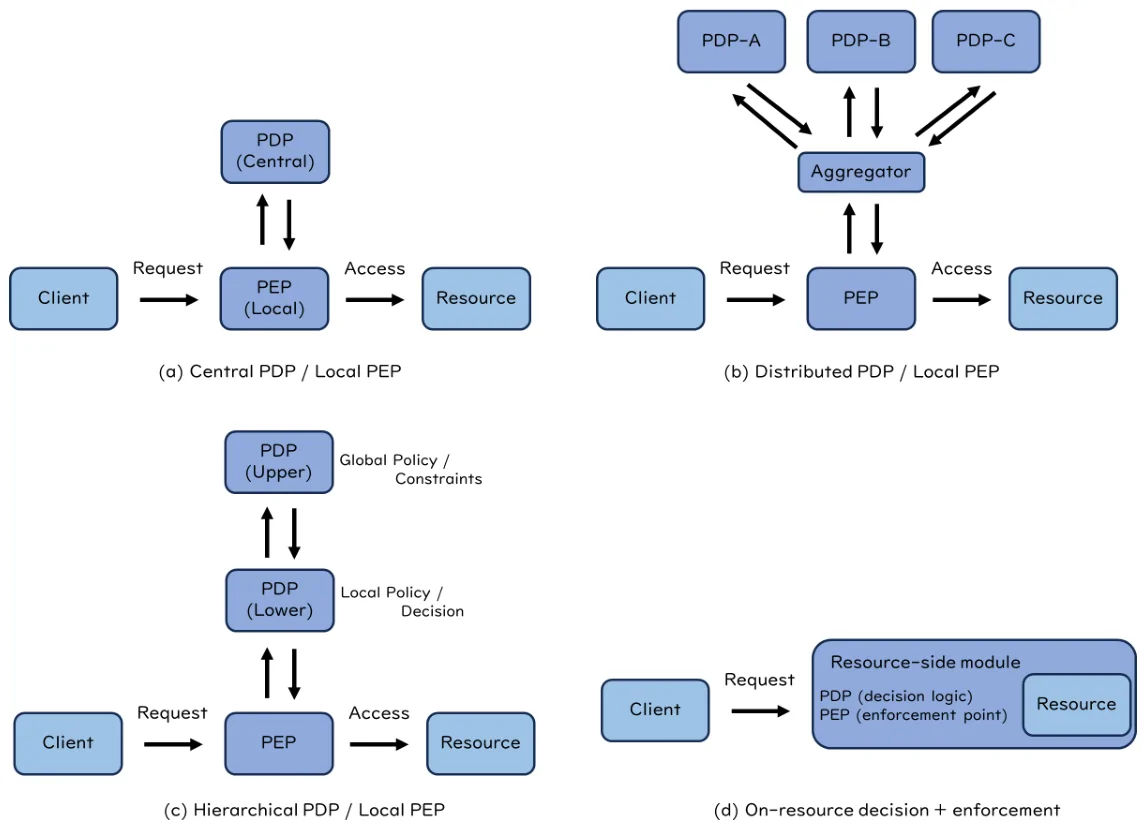
Four representative PDP–PEP architectural placement patterns used in the mapping.

**Figure 2:**
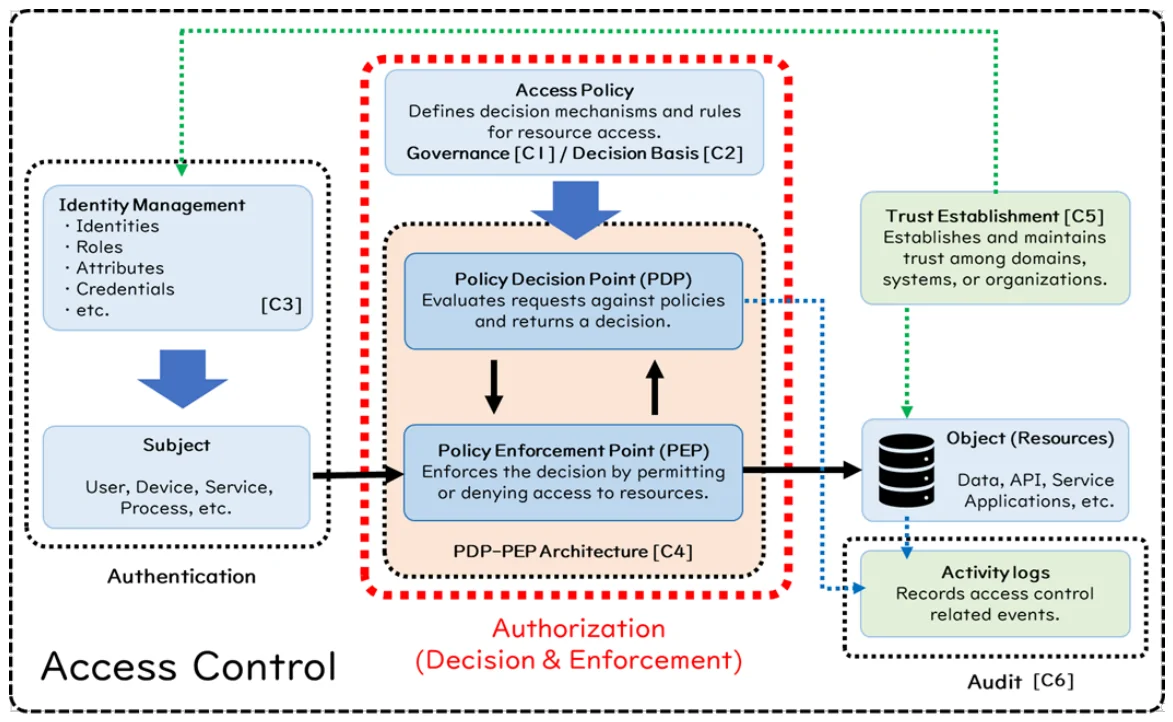
Conceptual relationship between access control components and the classification criteria C1–C6. Audit-related relationships are illustrative rather than exhaustive.

Figure 1 illustrates the four primary placement patterns used under C4, while Figure 2 shows how the six criteria relate to authorization-relevant components and information flows. The criteria are introduced here at a conceptual level. Their discrete labels, coding rules, and treatment of borderline cases are defined in Appendix C and applied to the selected studies in Section 4.4. The six criteria are summarized below.

- **Policy Governance (C1):** Captures where policy authority resides and who may define, update, delegate, or revoke authorization policies. The labels are Admin, Owner, Consortium, Hybrid, and Delegated.
- **Decision Basis (C2):** Captures the primary information or logic used to make authorization decisions. The labels are RBAC, ABAC, ReBAC, and Rule-based.
- **Identity and Attribute Management (C3):** Captures where and by whom authorization-relevant identities and attributes are issued, maintained, and obtained. The labels are Centralized, Federated, Distributed, and User-held.
- **PDP–PEP Architecture (C4):** Captures the placement and separation of authorization decision and enforcement functions. The primary patterns are Central PDP/Local PEP, Distributed PDP/Local PEP, Hierarchical PDP/Local PEP, and On-resource decision + enforcement, as illustrated in Figure 1. Additional architectural subtypes are recorded where applicable.
- **Trust Establishment (C5):** Captures the primary mechanism or trust root used to validate identities, attributes, credentials, policies, or authorization state across entities or domains. The labels include PKI-based trust, Federation trust, SSI (DID/VC), Consortium trust, Ledger-anchored integrity, and Local administrative trust.
- **Operational Assurance and Auditability (C6):** Captures explicitly reported mechanisms that support authorization-related logging, auditability, provenance, tamper evidence, and change control. Examples include Central logging, Tamper-evident log, Verifiable audit, and Policy change control.

To clarify how these criteria relate to the access control process, Figure 2 provides a conceptual mapping between the main access control components and the proposed classification criteria. The audit-related arrows in the figure are illustrative examples focused on authorization-related events; in practice, auditability may cover broader access-control-related events.

To complement criteria C1–C6, this study uses selected ZTA tenets as supplementary authorization-oriented analytical indicators. These indicators are not treated as primary classification criteria or as measures of overall ZTA compliance. For each selected study, correspondence with the authorization-relevant aspects of T3, T4, and T6 is coded using a three-level scale of **Yes**, **Partial**, or **No**.

- **Tenet 3 (T3):** Evaluates whether authorization is explicitly bounded to a session or validity interval through mechanisms such as leases, expiration, renewal, or reauthorization.
- **Tenet 4 (T4):** Evaluates whether authorization decisions incorporate dynamic policy inputs, including identity, device or application state, risk, location, and other contextual or environmental attributes.
- **Tenet 6 (T6):** Evaluates the authorization-related aspect of dynamic enforcement, including explicitly described reauthorization, revocation propagation, post-issuance validation, or policy-triggered re-evaluation.

These indicators concern authorization behavior and can therefore be applied to centralized, distributed, hierarchical, and resource-side architectures without assuming a particular physical deployment of ZTA components.

T1 and T2 are not used because they primarily concern resource scoping and communication protection, respectively. T5 and T7 concern asset-posture monitoring, telemetry, and security analytics. Although signals produced under T5 or T7 may serve as contextual inputs to T4 or trigger T6-related lifecycle actions, the mechanisms that generate those signals are outside the scope of the present coding. Authorization-related logging, auditability, provenance, and change-control mechanisms are recorded separately under C6.

Table 8 summarizes the use and scope of all seven ZTA tenets in this study.

### 4.3. Systematic Mapping Method

The study corpus was constructed through a systematic search and selection process designed to identify recent studies on authorization mechanisms in distributed environments.

This study follows the systematic mapping study (SMS) methodology described by Petersen et al. [56]. The primary objective of an SMS is to structure a research area by classifying the characteristics of selected studies and identifying recurring patterns, rather than to compare the effectiveness of individual proposals or synthesize their reported outcomes. Accordingly, the present study uses a predefined coding scheme to map architectural, governance, identity and attribute management, trust, and lifecycle characteristics across the selected corpus.

**Figure 3:**
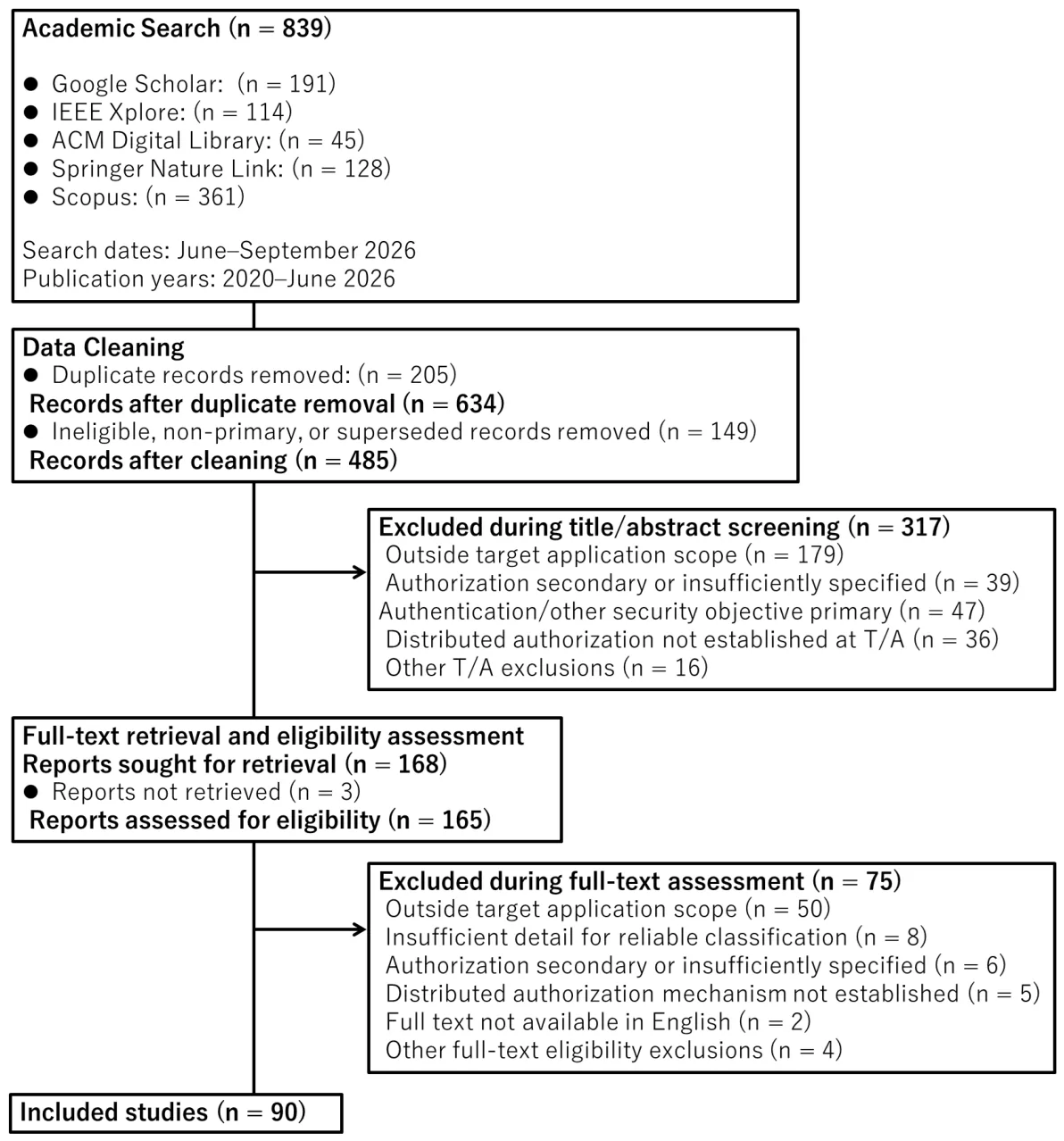
Overview of the literature search and study-selection process used to construct the mapping corpus.

The staged search, screening, and full-text eligibility process shown in Figure 3 is used to report study selection transparently. The classification criteria and coding rules are defined in Section 4.2 and Appendix C, while the coding procedure and inter-coder reliability assessment are described in Section 4.3.4.

To cover research in computer science, networking, and security, five academic search sources were used: Google Scholar, IEEE Xplore, ACM Digital Library, Springer Nature Link, and Scopus. The searches shared the broad conceptual focus of distributed or decentralized authorization and access control, but the platform-specific query formulations were not lexically identical. During revision, the Google Scholar search was rerun without the previous technology-specific blockchain/DLT/capability/token restriction, using the four phrases “distributed authorization”, “decentralized authorization”, “distributed access control”, and “decentralized access control”, combined using the OR operator. Scopus was also added using the same four broad application-neutral phrases. The previously completed IEEE Xplore, ACM Digital Library, and Springer Nature Link searches were retained using their respective platform-specific formulations. The exact search strings, execution dates, search-field settings where applicable, and retrieved record counts for each source are reported in Appendix A. The predefined application scope—IoT, edge and fog computing, sensor and WSN environments, and vehicular and V2X systems—was applied during study screening and eligibility assessment rather than as an additional application-specific restriction in the main search.

The main searches were conducted on 24 August 2026 for Google Scholar, 23 June 2026 for IEEE Xplore, 23 June 2026 for the ACM Digital Library, 22 June 2026 for Springer Nature Link, and 1 September 2026 for Scopus. A separate targeted sensitivity search using Relationship-Based Access Control (ReBAC) terminology, including “relationship-based access control”, “relationship-based authorization”, and “ReBAC”, was also conducted to assess potential retrieval bias associated with authorization-model-specific terminology. The results of this targeted search were cross-checked against the candidate studies identified through the main search process, and potentially eligible records were assessed using the same eligibility criteria.

For searches conducted after June 2026, records first made publicly available after 30 June 2026 were excluded to maintain a common publication window from 2020 through 30 June 2026. Annual distributions and year-based sensitivity analyses use the bibliographic publication year.

After applying the publication-period restriction, 839 records entered the study-selection process: 191 from Google Scholar, 114 from IEEE Xplore, 45 from the ACM Digital Library, 128 from Springer Nature Link, and 361 from Scopus. Platform-specific search strings, execution dates, publication-period restrictions, and retrieved record counts are reported in Appendix A. No backward or forward citation chasing (snowballing) was conducted; study identification was based on the predefined search-source queries and the supplementary sensitivity searches described above.

#### 4.3.1. Data Cleaning

After applying the predefined publication-period restriction, 839 records retrieved from Google Scholar, IEEE Xplore, the ACM Digital Library, Springer Nature Link, and Scopus were carried forward to data cleaning. All retained records were imported into Zotero and consolidated into a single library. Duplicate records arising from overlapping coverage among the search sources were identified primarily using exact DOI matches. When DOI information was unavailable, normalized-title matching was used, with author, publication year, and venue information examined in ambiguous cases.

Records with distinct non-empty DOIs were not merged solely based on title similarity. After duplicate removal, 634 unique records remained. The remaining records were then examined for publication type and record type.

Review and survey papers, white papers and technical reports, theses and dissertations, books and non-research book chapters, and other records that did not constitute original research studies were excluded at this stage. Original conference papers published in proceedings series were treated as research papers irrespective of whether their bibliographic metadata represented them as book chapters. Records affected by publication-integrity issues were also removed where applicable, and earlier versions that could be identified before screening as superseded by a more complete retained publication were excluded.

In total, 149 records were removed during this stage of data cleaning: 147 were ineligible publication types or other non-primary research records, one was removed because of a publication-integrity issue, and one was superseded by a more complete retained study. This left 485 records for title and abstract screening. Eligible research preprints were retained when they satisfied the same content and coding criteria as peer-reviewed publications, while relevant review articles were retained separately as background sources for the discussion of related work.

#### 4.3.2. Title and Abstract Screening

The 485 records retained after data cleaning were screened based on their titles and abstracts to identify studies whose primary contribution concerned authorization models or architectures in distributed environments within the predefined application scope.

Records were excluded when their primary application area fell outside the target scope, when authorization was secondary or insufficiently specified, when authentication or another security objective was primary, or when no qualifying distributed authorization mechanism or function could be established. At this stage, insufficient specification was used as an exclusion basis only when the title and abstract clearly indicated that the proposed authorization design itself lacked sufficient structural or semantic detail. Records for which eligibility could not be determined reliably from the title and abstract alone were advanced to full-text retrieval.

Each excluded record was assigned one primary exclusion reason to avoid double counting. A total of 317 records were excluded during title and abstract screening, leaving 168 reports for which full-text retrieval was sought, as summarized in Figure 3.

Title and abstract screening was conducted by a single reviewer (the first author) using the predefined eligibility criteria and was not independently double-screened. Consequently, no screening-specific inter-reviewer agreement statistic was calculated for this stage.

#### 4.3.3. Full-Text Assessment and Study Selection

Of the 168 reports for which full-text retrieval was sought after title and abstract screening, three could not be retrieved through the available institutional-access or open-access sources; the authors were not contacted individually to request copies. The remaining 165 reports were assessed for eligibility based on their full texts. The three reports that could not be retrieved were not assigned detailed C1–C6, ZTA, or other full-text-based classification codes.

For the full-text assessment, the application-domain scope comprised IoT, edge/fog, sensor/WSN, and vehicular/V2X environments and was applied consistently to all 165 reports assessed for eligibility.

At this stage, we examined whether each study provided sufficiently explicit descriptions of (i) the authorization model, including its decision basis and policy semantics, and (ii) the authorization architecture, including the placement and interaction of decision and enforcement components, to support coding under criteria C1–C6 and the selected ZTA indicators T3, T4, and T6.

The assessment focused on whether each study reported sufficient information for systematic mapping. It did not attempt to validate all performance claims, implementation details, or the technical effectiveness of the proposed mechanisms.

During full-text assessment, 75 reports were excluded, as summarized in Figure 3. Of these, 50 were excluded because their primary application area fell outside the predefined IoT, edge/fog, sensor/WSN, and vehicular/V2X scope. The remaining exclusions comprised studies with insufficient detail for reliable classification (*n* = 8), studies in which authorization was secondary or insufficiently specified (*n* = 6), studies for which a qualifying distributed authorization mechanism or function could not be established (*n* = 5), studies whose full text was not available in English (*n* = 2), and other full-text eligibility exclusions (*n* = 4). Each excluded report was assigned one primary exclusion reason to avoid double counting.

When multiple versions of the same study were identified, the most recent and complete eligible version was retained.

Detailed inclusion and exclusion criteria used during screening and eligibility assessment are provided in Appendix B.

Full-text eligibility assessment was likewise conducted by a single reviewer (the first author) using the predefined inclusion and exclusion criteria. This stage was not independently double-screened, and therefore no separate inter-reviewer agreement statistic was calculated for full-text study selection. Eligibility decisions and primary exclusion reasons were recorded to support auditability of the selection process.

After applying these criteria, 90 studies were retained as the final mapping corpus (*studies included in the mapping* in Figure 3). These studies form the analytical basis for the mapping and synthesis presented in the following sections.

**Figure 4:**
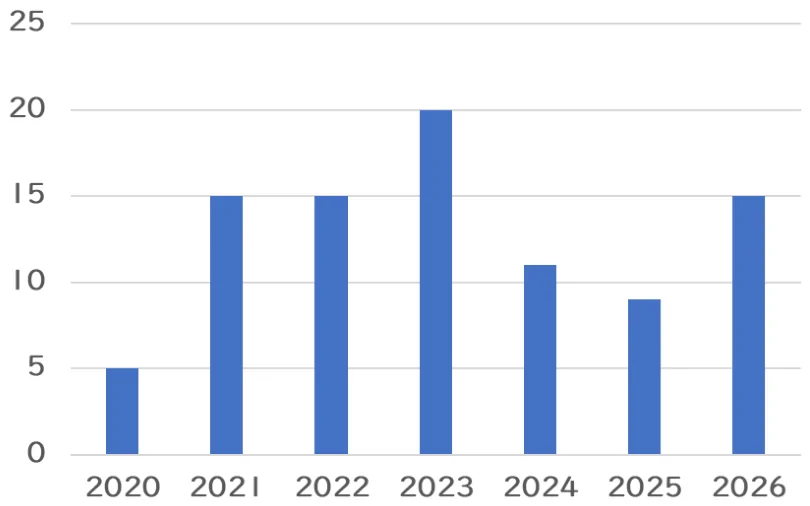
Distribution of selected studies by publication year; the 2026 count covers studies available through June 2026.

Figure 4 and Table 9 summarize the final mapping corpus by publication year and publisher/platform, respectively. The largest number of selected studies was published in 2023, and IEEE publications constituted the largest publisher/platform category. Publisher/platform categories are reported rather than search sources because individual studies may be indexed by more than one search source.

#### 4.3.4. Coding Procedure and Inter-Coder Reliability

The primary coder (the first author) conducted the full-text coding of all 90 included studies using the predefined criteria and coding rules described in Section 4.2 and Appendix C. To assess coding reproducibility, a subset of 20 studies (22.2% of the final corpus) was independently blind-coded by a second coder using the same classification codebook and coding instructions. Eligibility, application scope, and language availability were not evaluated as part of this reliability assessment; the assessment focused on the classification coding of the included studies.

For the single-label categorical variables C1, C2, C3, C4 Primary, C5, and DLT/SC use, percentage agreement and Cohen’s kappa were calculated. For the ordinal ZTA indicators T3, T4, and T6, percentage agreement and linear-weighted Cohen’s kappa were calculated. For the multi-label variables C4 subtypes, C6, and DLT/SC roles, agreement was assessed using exact-set agreement and mean Jaccard similarity, because these fields can contain multiple simultaneously applicable labels.

For the single-label variables, percentage agreement was 70.0% for C1 (κ=0.574), 85.0% for C2 (κ=0.748), 80.0% for C3 (κ=0.696), 85.0% for C4 Primary (κ=0.776), 70.0% for C5 (κ=0.586), and 100.0% for DLT/SC use (κ=1.000). For the multi-label fields, C4 subtypes showed an exact-set agreement of 26.3% and a mean Jaccard similarity of 0.669 (n=19), C6 showed an exact-set agreement of 50.0% and a mean Jaccard similarity of 0.667 (n=20), and DLT/SC roles showed an exact-set agreement of 7.7% and a mean Jaccard similarity of 0.764 (n=13). The two reduced denominators have different causes: for C4 subtypes, the field was left blank by the second coder for one study, and this entry was treated as missing rather than as an empty label set; for DLT/SC roles, the field is conditional on DLT/SC use, so agreement was computed only over the 13 studies that both coders coded as using DLT/SC. For the ZTA indicators, percentage agreement was 85.0% for T3 (linear-weighted κ=0.851), 65.0% for T4 (linear-weighted κ=0.515), and 70.0% for T6 (linear-weighted κ=0.054). Detailed variable-level reliability results and coder-level comparisons are provided in the Supplementary Materials.

The multi-label results show that the exact-set criterion was substantially stricter than the label-overlap criterion, particularly for C4 subtypes and DLT/SC roles, where the two coders often identified overlapping but not identical sets of applicable labels. The notably low linear-weighted kappa for T6, despite a percentage agreement of 70.0%, reflects the strongly skewed marginal distributions of this indicator in the reliability sample: the primary coder assigned Yes to 16 studies and Partial to 4, whereas the second coder assigned Yes to 16, Partial to 3, and No to 1. Because both coders’ marginal distributions were strongly concentrated in the Yes category, the expected agreement under chance was high, resulting in a low chance-corrected coefficient despite relatively few observed disagreements. Five of the six disagreements occurred at the adjacent Yes/Partial boundary, with only one Yes/No disagreement. T6 was therefore interpreted using both percentage agreement and linear-weighted kappa rather than the kappa value alone, and the corresponding coder-level disagreement pattern is reported in the Supplementary Materials.

All reliability statistics reported above were calculated before reconciliation, from the two independently produced coding sets. After the reliability assessment, disagreements in the 20-study subset were reviewed against the full texts and reconciled by the two coders, including the final assignment of primary and secondary labels where applicable. Reconciliation did not alter the classification framework or label sets, but clarified several operational coding boundaries. Following reconciliation, the primary coder conducted a corpus-wide consistency review of all 90 included studies using these clarified boundaries. Studies outside the 20-study reliability subset were not independently recoded by the second coder. The final corpus-level coding was subsequently reviewed by the co-authors. Any final-label updates resulting from reconciliation and the subsequent corpus-wide consistency review are reflected in the study-level coding matrix in Supplementary Table S1. The reliability statistics, however, remain based exclusively on the original pre-adjudication coding of the 20-study subset and were not recalculated after reconciliation.

### 4.4. Classification of Selected Distributed Authorization Studies

This subsection applies criteria C1–C6, introduced in Section 4.2, to the 90 selected studies. The application-area labels reported in Table 10, Table 11 and Table 12 are treated as descriptive study attributes rather than as an additional analytical dimension of the taxonomy.

The purpose of the mapping is not to rank the technical quality or effectiveness of the proposed models. Instead, it provides a consistent analytical view of how the selected studies differ in policy governance, decision basis, identity and attribute management, PDP–PEP architectural placement, trust establishment, and operational assurance and auditability. Each study was assigned discrete labels under the predefined coding rules. Study-specific details and borderline judgments were recorded during the coding process.

The complete label definitions, coding rules, and treatment of borderline cases are provided in Appendix C. The coding procedure and inter-coder reliability assessment are described in Section 4.3.4.

**Notes on coding CAP-BAC and C6**

Before presenting the mapping results, we clarify the treatment of CAP-BAC and C6, which may otherwise be interpreted inconsistently across studies.

First, CAP-BAC is treated as a credential-carrying authorization pattern in which a capability represents an authorization grant that can be independently validated by the enforcement side. By contrast, Token-based denotes the broader use of tokens to convey authorization-related information and does not by itself imply CAP-BAC. The two labels are therefore not mutually exclusive: a capability may be implemented as a token, whereas general token-based authorization may still depend on issuer-mediated validation or may not represent an independently verifiable authorization grant.

CAP-BAC is recorded as a C4 architectural subtype rather than as a C2 decision-basis category because it characterizes how an authorization grant is represented, conveyed, and independently validated at the enforcement side. The logic used to determine whether that grant should be issued, such as RBAC, ABAC, ReBAC, or rule-based logic, is recorded separately under C2. C4 records the placement of authorization decision and enforcement functions and captures CAP-BAC as an additional architectural characteristic when independently verifiable capability grants are used. Study-specific interpretations and borderline cases are documented in the coding notes.

Second, C6 records explicitly reported mechanisms supporting operational assurance and auditability. The predefined C6 labels are Central logging, Tamper-evident log, Verifiable audit, and Policy change control. C6 is treated as a multi-label field, and multiple labels are assigned when more than one corresponding mechanism is explicitly reported in a study. C6 does not define the authorization decision basis or the primary PDP–PEP architectural pattern. Instead, it is used as a complementary dimension for mapping the operational-assurance and auditability mechanisms explicitly described in each study. An em dash indicates that no corresponding C6 mechanism was explicitly reported.

Based on this coding scheme, Table 10, Table 11 and Table 12 present the study-level mapping results for the 90 selected studies. Application area is reported as a descriptive attribute using four labels: IoT, Edge, WSN, and V2X. To support comparison across the three tables, the studies are presented in an identical order based on application area, publication year, and first-author name.

For readability, the results are divided as follows: Table 10 reports application area and the coded values for C1–C3, C5, and C6; Table 11 reports application area, C4 architectural placement patterns, and C4 subtypes; and Table 12 reports application area, correspondence with the authorization-relevant aspects of the selected ZTA tenets T3, T4, and T6, and the reported use and functional roles of DLTs and SCs. All applicable DLT/SC functional roles are retained as multi-label coding without imposing a fixed maximum number of role tags.

#### 4.4.1. Overall Distribution of C1–C6 Classification Results

Table 13 summarizes the overall distributions of the six classification criteria across the 90 selected studies.

##### PDP–PEP Architectural Placement (C4)

**Figure 5:**
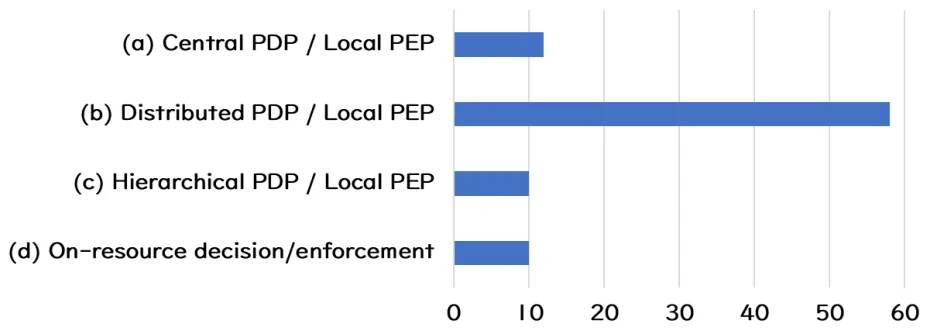
Distribution of the selected studies by PDP–PEP architectural placement pattern (C4).

C4 captures the placement and separation of authorization decision and enforcement functions, but does not by itself determine whether the overall authorization design is distributed or decentralized. Table 13 and Figure 5 show that Distributed PDP/Local PEP is the dominant C4 pattern in the mapped corpus; this prevalence implies neither technical superiority nor decentralized policy authority.

Central PDP/Local PEP centralizes the substantive authorization decision while keeping enforcement close to the protected resource, although identity, trust, credential validation, or policy-related state may still be distributed. Distributed PDP/Local PEP uses multiple or replicated decision functions with local enforcement and is common in DLT/SC-assisted designs, but C1, C3, and C5 remain independent dimensions. Hierarchical PDP/Local PEP combines upper-level policy or coordination functions with lower-level decision, validation, or enforcement functions. On-resource decision + enforcement co-locates the substantive Grant/Deny decision and enforcement at the protected resource; local verification of a grant already determined by an upstream PDP does not by itself qualify as On-resource decision making. Thus, C4 placement must be interpreted together with the other classification dimensions rather than as a stand-alone measure of decentralization.

##### Decision Basis (C2)

**Figure 6:**
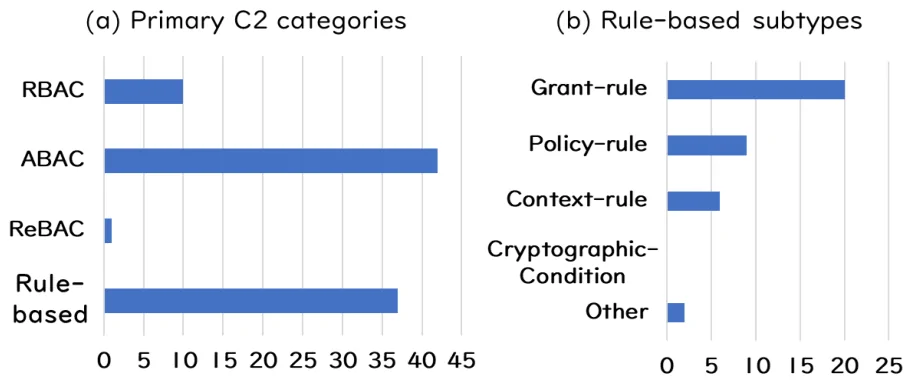
Distribution of authorization decision-basis categories (C2) across the selected studies: (**a**) primary C2 categories and (**b**) Rule-based subtypes of the 37 studies whose primary C2 category is Rule-based.

Figure 6 shows the primary C2 categories and the predefined subtypes of studies coded as Rule-based. RBAC, ABAC, and ReBAC are assigned only when the corresponding access control model is explicitly instantiated; otherwise, mechanisms expressed primarily through rules or conditional logic are coded as Rule-based. ABAC and Rule-based authorization account for most of the corpus, while RBAC and especially ReBAC are less frequent.

Because CAP-BAC is coded as a C4 subtype, Table 14 also reports an alternative C2 convention in which the 27 CAP-BAC studies form a separate category; 20 of these studies are Rule-based and seven are ABAC under the primary scheme. Under this alternative, ABAC remains the largest C2 category while Rule-based decreases, supporting the distinction between decision semantics and capability-based grant representation.

The 37 Rule-based studies were further characterized as Grant-rule (20), Policy-rule (9), Context-rule (6), or Other (2); no study was assigned the Cryptographic-condition subtype. ReBAC is observed in only one selected study, Polychronaki et al. [94], and a dedicated ReBAC sensitivity search identified no additional eligible studies within the predefined scope. This limited representation is therefore treated as a characteristic of the mapped corpus rather than as evidence that ReBAC is unsuitable for distributed environments.

##### Distribution of C5 (Trust Establishment)

Ledger-anchored integrity is the most frequent primary C5 label, while Consortium, Local administrative, SSI, Federation, and PKI-based trust are also represented; material secondary mechanisms are retained for the sensitivity analysis below. The distribution shows that distributed authorization does not imply a single trust model: ledger-based integrity, organizational or consortium relationships, federated trust, SSI credentials, and PKI can each underpin authorization-relevant trust. In particular, Ledger-anchored integrity denotes the ledger’s role as an integrity anchor and does not by itself imply decentralized governance or complete lifecycle control.

##### Distribution of C1 (Policy Governance)

Owner and Admin governance account for most primary C1 classifications, with smaller Hybrid, Delegated, and Consortium groups; secondary governance characteristics are retained separately. The distribution confirms that policy authority is independent of PDP–PEP placement: distributed decision or enforcement functions may coexist with owner- or administrator-controlled policy, while other studies share, combine, or delegate policy authority. Accordingly, architectural distribution should not be used as a proxy for decentralized governance.

##### Distribution of C3 (Identity and Attribute Management)

Distributed identity and attribute management is the most frequent primary C3 arrangement, but Centralized, Federated, and User-held approaches are also represented and secondary arrangements are retained separately. Thus, a distributed authorization architecture may still rely on a single identity authority, while other designs use cross-domain federation, multiple authorities or registries, or holder-mediated credentials. C3 is therefore interpreted independently of decision placement, policy governance, and trust establishment.

##### Observation on C6 (Operational Assurance and Auditability)

C6 is multi-label and records all explicitly reported authorization-related assurance and auditability mechanisms. Tamper-evident log is the most frequent C6 mechanism, followed by Policy change control and Verifiable audit; Central logging is rare, and 12 studies report none of the predefined C6 mechanisms. Tamper evidence alone does not imply independently verifiable audit, and Policy change control is distinguished from credential, token, or capability revocation, which is treated as lifecycle handling. C6 is descriptive of reported assurance mechanisms and is not used as a measure of technical maturity or overall system quality.

#### 4.4.2. Correspondence with Selected ZTA Tenets and DLT/SC Usage

##### Correspondence with Selected ZTA Tenets (T3, T4, and T6)

Table 15 reports correspondence with the authorization-relevant aspects of T3, T4, and T6. T6 has the highest Yes proportion (70.0%), while T3 and T4 are each coded Yes in approximately half of the corpus; 20 studies are coded Yes for all three tenets (see Table 12). A Partial result indicates that a relevant mechanism is described but not specified comprehensively or explicitly enough for a Yes classification. T3 = Yes requires explicit bounded authorization validity, T4 = Yes requires a changing current value to affect the Grant/Deny decision, and T6 = Yes requires request-time or current-state validation, reauthorization, revocation/status checking, or another lifecycle change capable of affecting subsequent access. These results measure correspondence with selected authorization-relevant ZTA aspects rather than overall ZTA compliance, technical quality, or system maturity.

##### DLT/SC Usage

DLTs and/or SCs are reported in 70 of the 90 selected studies (77.8%; Table 12), supporting functions such as policy evaluation, shared policy state, trust anchoring, audit logging, governance, coordination, and authorization-state management. When a ledger is the primary integrity or trust anchor for authorization-relevant information, this role is also reflected under C5 as Ledger-anchored integrity.

##### Functional Roles of DLT/SC Usage

**Figure 7:**
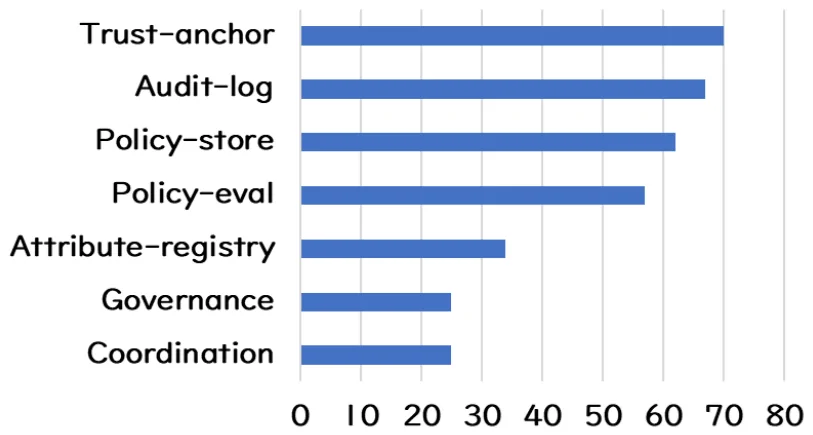
Distribution of reported functional roles of DLTs and SCs in the selected studies. Of the 90 selected studies, 70 report DLT and/or SC usage and 20 do not. All applicable functional tags are retained for each DLT/SC-assisted study, and multiple tags may therefore be assigned to a single study.

All applicable DLT/SC role tags defined in Appendix C.10 are retained without a fixed upper limit. Among the 70 DLT/SC-assisted studies, Trust-anchor appears in 70, Audit-log in 67, Policy-store in 62, Policy-eval in 57, Attribute-registry in 34, and Governance and Coordination in 25 each; these multi-label counts are not mutually exclusive. Figure 7 shows that ledger use extends beyond trust anchoring to auditability, shared policy state, policy evaluation, identity-related registries, governance, and coordination.

##### Three Recurrent Usage Patterns

For interpretive synthesis, the multi-label DLT/SC roles can be grouped into three overlapping patterns that are not additional coding categories or statistically derived clusters. Decision and state-management substrate usage combines Policy-eval and Policy-store (53/70), often with Coordination, to support shared execution or authorization state while enforcement may remain local or off-chain. Trust and registry infrastructure usage combines Trust-anchor and Attribute-registry (34/70) to provide shared integrity references for authorization-relevant identity or attribute information without determining which issuers or claims should be trusted. Assurance, provenance, and governance support combines Audit-log and Governance (24/70) to record authorization-relevant events or state changes even when evaluation and enforcement occur elsewhere. Because these patterns overlap, a single study may support several of them simultaneously.

#### 4.4.3. Application-Domain-Stratified Analysis

The final corpus comprises 67 IoT, 16 Edge, 4 WSN, and 3 V2X studies, so aggregate results are influenced strongly by the IoT subset. The following comparisons therefore use within-domain percentages and are descriptive rather than tests of statistically significant differences. The WSN and V2X subsets are too small for broad domain-level generalization and are treated as exploratory.

##### Domain-Level Variation in C1–C6

C1–C5 are compared using primary labels and C6 using all applicable labels (Table 10 and Table 11). The two larger subsets show the clearest contrasts: Owner governance and ABAC are most frequent in IoT, whereas Admin governance and Rule-based authorization dominate Edge; Distributed PDP/Local PEP is also more prevalent in IoT (68.7%) than Edge (43.8%). Distributed C3 and Ledger-anchored C5 are the largest categories in both domains, but Edge shows a broader mixture of identity, trust, and C4 arrangements, while Tamper-evident logging is more common in IoT (82.1%) than Edge (50.0%). These differences indicate that the aggregate C1–C6 distributions are not uniform across application areas and should be interpreted with the corpus imbalance in mind.

##### Domain-Level Variation in Selected ZTA Tenets and DLT/SC Usage

T3 and especially T6 show broadly similar Yes proportions in the IoT and Edge subsets, whereas T4 is lower in Edge. A clearer contrast appears in DLT/SC usage, reported in 58/67 IoT studies (86.6%) but 8/16 Edge studies (50.0%), indicating that the high corpus-wide prevalence of DLT/SC-assisted authorization is driven particularly by IoT. The smaller WSN and V2X subsets again remain exploratory rather than evidence of stable domain-level differences. Detailed domain-normalized distributions of C1–C6, T3, T4, T6, and DLT/SC usage are provided in Supplementary Table S4.

#### 4.4.4. Cross-Dimensional Relationships

##### Relationship Between Policy Governance (C1) and PDP–PEP Placement (C4)

Among the 58 Distributed PDP/Local PEP studies, 29 use Owner and 18 use Admin governance, with the remainder distributed across Hybrid, Consortium, and Delegated governance. Because Owner and Admin governance also occur across the other C4 patterns, decision placement does not uniquely determine policy authority.

##### Relationships of C4 with Identity and Attribute Management (C3) and Trust Establishment (C5)

Among the 58 Distributed PDP/Local PEP studies, Distributed is the most frequent C3 label (34/58) and Ledger-anchored integrity the most frequent C5 label (36/58), but substantial minorities use other identity and trust arrangements. The other C4 patterns are similarly heterogeneous, supporting treatment of decision placement, identity management, and trust establishment as distinct dimensions.

##### Relationship Between C4 Placement and Selected ZTA Tenets

Table 16 compares the proportion of studies coded as Yes for the authorization-relevant aspects of T3, T4, and T6 across the four C4 architectural placement patterns.

The comparison shows that correspondence with the selected ZTA tenets varies across C4 patterns rather than following a single decentralization gradient. T4 is highest in the Hierarchical pattern, while T6 = Yes is common in Central, Distributed, and Hierarchical patterns but lower for On-resource designs, where five of ten studies are T6 = Partial; T3 varies less strongly. Thus, PDP–PEP placement alone does not determine session-bounded access, dynamic policy inputs, or dynamic authorization enforcement.

##### Relationship Between DLT/SC Roles and C4 Architectural Placement

DLT/SC use is most prevalent in Distributed PDP/Local PEP (54/58) and Hierarchical PDP/Local PEP (9/10) studies, compared with Central (3/12) and On-resource (4/10) patterns. Distributed PDP studies frequently combine Policy-store, Policy-eval, and Audit-log roles, whereas Hierarchical designs show relatively more Coordination and Governance and On-resource designs use the ledger mainly for external trust, registry, or assurance functions rather than local Policy-eval. These differences are functional rather than deterministic: neither DLT/SC use nor a particular ledger role uniquely determines decision or enforcement placement.

##### Relationship Between DLT/SC Usage and T6 Dynamic Enforcement

T6 = Yes occurs in 70.0% of both the DLT/SC-assisted and non-DLT groups, indicating that ledger use is neither necessary nor sufficient for strong T6 correspondence.

##### Additional Cross-Dimensional Observations

Distributed PDP/Local PEP studies span ABAC, Rule-based, RBAC, and ReBAC decision bases, while Owner governance occurs with all four C3 arrangements. Likewise, identity-management and trust labels form multiple combinations, reinforcing that governance, decision basis, identity management, architectural placement, and trust establishment are related but non-interchangeable dimensions. Detailed cross-tabulation matrices are provided in Supplementary Table S4.

#### 4.4.5. Study Maturity and Evaluation Characteristics

Study maturity and evaluation setting are recorded separately: the former describes development stage, whereas the latter describes the environment in which a proposal was evaluated.

##### Publication Status and Study Maturity

Of the 90 studies, 87 are peer-reviewed and 3 are preprints; 85 are classified as Evaluated implementation, 3 as Conceptual, and 2 as Prototype. No study is classified as a Real-world deployment. Thus, implementation-based evidence is common, but sustained operational-deployment evidence is absent; these maturity categories are descriptive rather than quality scores.

##### Threat-Model Reporting

Explicit threat models are reported by 43 studies, partial threat assumptions by 36, and no clear threat model by 11, indicating that threat-model specification remains uneven across the corpus.

##### Reproducibility

Public artifacts or sufficiently detailed experimental resources are available for 25 studies, partial reproducibility information for 60, and little or none for 5, so fully accessible artifacts remain available for only about one quarter of the corpus.

##### Evaluation Setting

Prototype/Testbed settings dominate (59 studies), followed by Mixed (17) and Simulation (8), with 3 Theoretical and 3 Not reported, indicating that most empirical evidence comes from controlled rather than operational environments.

##### Performance, Scalability, and Deployment Evidence

Performance and scalability are reported using heterogeneous metrics and experimental configurations, including latency, throughput, computation or communication overhead, and scaling with devices, users, or authorization requests. Because these measures are reported under substantially different platforms, workloads, and system assumptions, they are not pooled into a common quantitative comparison. Together with the absence of Real-world deployment classifications, this heterogeneity supports interpreting the corpus primarily as evidence of technical feasibility rather than sustained operational effectiveness.

#### 4.4.6. Robustness and Sensitivity Analyses

Two sensitivity analyses assess whether the principal interpretations depend on primary-only coding or corpus composition.

##### Sensitivity to Secondary Classifications

Secondary labels occur in 24/90 studies for C1, 19/90 for C2, 38/90 for C3, and 48/90 for C5, but no dominant primary category is displaced when they are included. The main change is greater visibility of mixed mechanisms, particularly in C3 and C5: User-held rises from 11 primary labels to 25 primary-or-secondary occurrences, Local administrative trust from 15 to 26, and PKI-based trust from 3 to 12. Detailed secondary classifications are provided in the Supplementary Materials.

##### Sensitivity to Corpus Composition

Excluding preprints (n=87), 2026 studies (n=75), or both (n=73) leaves the dominant C1, C2, C3, and C5 categories unchanged; Distributed PDP/Local PEP remains at 64.4–65.8%, DLT/SC usage at 77.0–81.3%, and T6 = Yes at 69.3–71.3%. Thus, the principal corpus-level interpretations are not materially driven by preprints or the partial 2026 publication year; detailed distributions are provided in the Supplementary Materials.

### 4.5. Synthesis: Relating Architectural Choices to Selected ZTA Tenets

The preceding analyses show that distributed authorization is better understood as a combination of multiple design dimensions rather than as a single architectural property. In particular, distributed PDP placement does not necessarily imply distributed policy governance, identity and attribute management, or trust establishment. Similar placement patterns occur with different governance and trust arrangements, indicating that C1–C6 capture related but distinct design choices. This distinction is important because architectures that appear similarly distributed at the decision layer may differ substantially in where authority, identity management, trust, and enforcement reside.

Taken together, the DLT/SC analyses show that ledger-assisted mechanisms support multiple authorization functions, while the governance, architectural-placement, trust, and authorization-lifecycle properties captured by the taxonomy remain analytically distinct from DLT/SC use. This interpretation is reinforced by the identical observed proportion of T6 = Yes classifications (70.0%) in the DLT/SC-assisted and non-DLT groups.

More generally, correspondence with the selected ZTA tenets T3, T4, and T6 depends on combinations of authorization design choices rather than on a particular architecture or technology. Session-bounded access, dynamic policy inputs, and dynamic enforcement depend on how credential validity, contextual information, policy changes, revocation, status validation, and enforcement behavior are integrated. The mapping therefore suggests that distributed authorization and ZTA correspondence should be assessed across multiple architectural and lifecycle dimensions rather than inferred from distribution or technology choice alone.

### 4.6. Limitations and Future Research Directions

#### 4.6.1. Limitations of the Present Study

This systematic mapping study has several limitations.

First, the classification is based on information explicitly reported in the selected studies. Architectural, governance, trust, assurance, and lifecycle mechanisms that were implemented but not sufficiently described could therefore not be fully reflected in the assigned labels. This limitation is particularly relevant to T3, T4, and T6, for which conservative coding rules were applied when the required authorization behavior was not explicitly established. Accordingly, a No or Partial classification does not necessarily indicate that the corresponding mechanism is absent from the implemented system, but rather that sufficient evidence was not available in the publication.

Second, this study maps reported design characteristics rather than providing a comparative assessment of overall technical quality or security effectiveness. Study maturity, threat-model reporting, reproducibility, and evaluation setting were recorded descriptively, but no common quality-scoring or risk-of-bias instrument was applied across the corpus. Performance and scalability results were also reported under heterogeneous platforms, workloads, metrics, and experimental conditions and were therefore not pooled into a common quantitative comparison. The reported frequencies should consequently be interpreted as characteristics of the mapped literature rather than as rankings of architectural or technical effectiveness.

Third, the final corpus remains bounded by the selected search sources, publication period, terminology, and search formulations. To reduce potential search bias, the revision included a technology-neutral Google Scholar search without the previous blockchain/DLT/capability/token restriction, the addition of a broad application-neutral Scopus search, and a targeted ReBAC sensitivity search. The previously completed IEEE Xplore, ACM Digital Library, and Springer Nature Link searches were retained using their respective platform-specific formulations, and the search queries were therefore not lexically identical across all five sources. This remaining query heterogeneity may have affected retrieval and is a limitation of the present study. No backward or forward citation chasing (snowballing) was conducted, so eligible studies not captured by the search queries may have been missed. Coverage may also be limited by differences in terminology and indexing. In addition, the Google Scholar search was pragmatically limited to the first 10 relevance-ranked result pages. Because the complete result set was not screened, eligible studies ranked below this retrieval cutoff may have been missed. The prevalence of particular mechanisms, including DLT/SC-assisted authorization, should therefore be interpreted as a characteristic of the selected corpus rather than as an estimate of the entire distributed authorization literature. Sensitivity analyses excluding preprints and studies from the partial 2026 publication year indicated that the principal corpus-level patterns were stable, although these analyses do not eliminate the remaining coverage limitations.

Fourth, the complete corpus was not independently coded by multiple reviewers. The primary classification was conducted by the first author, while 20 of the 90 studies (22.2%) were independently blind-coded by a second coder and inter-coder agreement was assessed before reconciliation. Agreement was substantial for several dimensions but varied across categories and was affected by marginal imbalance for some measures, particularly T6. The remaining 70 studies were coded by the primary coder under the same predefined coding rules. The reliability exercise provides quantitative evidence regarding coding reproducibility, but it also indicates that some coding boundaries remain difficult to apply consistently. In particular, T6 showed low chance-corrected agreement despite 70.0% raw agreement, with most disagreements occurring at the Yes/Partial boundary. The corpus-level T6 distribution should therefore be interpreted with this residual coding uncertainty in mind. Full duplicate coding of all 90 studies would provide stronger reliability evidence.

Finally, the application-domain scope of the mapping is limited to IoT, edge/fog computing, sensor/WSN environments, and vehicular/V2X systems, and the corpus is unevenly distributed across these domains. IoT accounts for 67 of the 90 studies, compared with 16 Edge, 4 WSN, and 3 V2X studies. Domain-normalized analyses were used to reduce the effect of this imbalance, but the small WSN and V2X subsets do not support broad domain-level generalization. The taxonomy may also be applicable to other distributed settings, such as multi-cloud systems, data spaces, decentralized applications, and cross-organizational service ecosystems, but these domains were not systematically covered in the present study.

#### 4.6.2. Research and Design Directions

Several research directions emerge from the mapping results.

First, distributed authorization proposals should specify authorization-lifecycle behavior more explicitly. Many studies describe request-time policy evaluation, credential or token validation, and access enforcement more clearly than subsequent lifecycle behavior. Future designs should therefore specify re-evaluation triggers, revocation and policy-change propagation, post-issuance validation, and the responsibilities of PDPs and PEPs when identity, attribute, risk, or environmental state changes. Such specifications would also clarify correspondence with T3, T4, and T6.

Second, policy governance, identity and attribute management, PDP–PEP placement, and trust establishment should be treated as separate but coordinated design dimensions. The cross-dimensional analysis shows that distributing decision functions does not determine who controls policies, which entities issue authorization-relevant information, or which trust anchors are accepted. Future multi-domain systems should therefore make policy ownership, delegation authority, identity and attribute sources, trust relationships, and administrative revocation responsibilities explicit.

Third, relationship- and delegation-oriented authorization remains sparsely represented in the mapped literature. In particular, ReBAC appears only rarely compared with ABAC and Rule-based approaches. Further research is needed on mechanisms that explicitly represent relationships, delegation chains, authority attenuation, and changes in relationship state, especially when authorization authority must propagate across organizations, services, devices, and users.

Fourth, semantic and operational interoperability require greater attention. Exchanging credentials, attributes, policies, or authorization state does not ensure that participating domains interpret them consistently. Future work should therefore address heterogeneous attribute vocabularies, policy semantics, credential representations, assurance levels, and mechanisms for detecting and resolving conflicting policies or inconsistent authorization state.

Fifth, evaluation should extend beyond request-time latency and cryptographic cost. Future studies should examine revocation latency, policy-update propagation, reauthorization behavior, coordination overhead, scalability across administrative domains, failure handling, and recovery from inconsistent authorization state. More evaluations in realistic and operational environments are also needed, because the present corpus is dominated by prototype, testbed, simulation, and mixed experimental settings rather than real-world deployments. Comparative evaluation of DLT/SC-assisted and non-DLT designs would further help distinguish properties of the authorization model from those of the implementation substrate.

##### Design Implications

Taken together, the findings suggest that distributed authorization systems should document policy-governance responsibility, identity and attribute sources, PDP–PEP placement, trust assumptions, and operational-assurance mechanisms independently. DLT/SC-assisted designs should additionally distinguish functions performed on-chain from those performed off-chain, while token- and capability-based designs should make grant scope, delegation, validity, status checking, renewal, and revocation explicit. These practices would make architectures with different distribution and implementation strategies easier to compare and evaluate consistently.

## 5. Conclusions

This paper developed a standards-informed taxonomy and systematic mapping framework for distributed authorization and applied it to 90 studies from IoT, edge/fog, sensor/WSN, and vehicular/V2X environments. The framework distinguishes six dimensions covering policy governance, decision basis, identity and attribute management, PDP–PEP architecture, trust establishment, and operational assurance and auditability.

Within the mapped corpus, Distributed PDP/Local PEP was the dominant architectural pattern, appearing in 58 studies, while ABAC and Rule-based mechanisms were the most common decision bases. However, distributed decision placement did not necessarily imply decentralized policy governance, identity and attribute management, or trust establishment. DLTs and/or SCs were reported in 70 studies, but their use likewise did not by itself establish decentralization or complete authorization-lifecycle control.

The analysis using the selected ZTA tenets further showed that authorization-lifecycle mechanisms are widely represented, although their specification remains incomplete in part of the literature. Overall, the results support treating distributed authorization as a combination of related but distinct design dimensions. Future work should strengthen lifecycle behavior, cross-domain interoperability, relationship- and delegation-oriented authorization, and real-world operational evaluation.

## Figures and Tables

**Table 1:** Comparison of prior surveys with the analytical scope of the present study.

Survey	Search Window	Gov	DB	IDM	PP	Tr	AL	ZTA	DLT	Main Distinction from This Study
Mohammad [13]	No date limit	P	E	P	–	E	P	–	–	Cloud access control: requirements and mechanisms rather than architectural placement and lifecycle analysis.
Mohamed et al. [14]	Not stated	E	E	–	–	–	–	–	–	General authorization: definitions, strategies, and model classification rather than distributed placement.
Badirova et al. [15]	Recent years (exact range not stated)	E	P	E	–	E	P	–	E	Cross-domain IAM: federation, decentralized identity, and revocation rather than decision–enforcement placement.
Uppuluri and Lakshmeeswari [16]	Not stated	–	P	P	–	P	–	–	P	IoT smart homes: security and privacy techniques rather than cross-domain governance and architecture.
Wijesekara [17]	Past decades (exact range not stated)	P	E	P	P	P	P	–	E	Blockchain-based networking: ledger-assisted techniques rather than technology-neutral authorization dimensions.
Babu and Suresh Babu [18]	Not stated	P	E	–	–	P	–	–	E	Blockchain access control: model comparison and challenges rather than standards-informed multidimensional coding.
Pérez Díaz and Almenares Mendoza [19]	After 2013	P	E	P	P	P	P	–	P	IoT authorization: models, platforms, and architectures rather than jointly coded governance, trust, and lifecycle.
**This study**	**2020–June 2026**	**E**	**E**	**E**	**E**	**E**	**E**	**E**	**E**	Joint C1–C6 coding across IoT, edge/fog, WSN, and V2X, with selected ZTA correspondence and functional DLT/SC role analysis.

*Note:* Gov = policy governance; DB = decision basis; IDM = identity and attribute management; PP = PDP–PEP architectural placement; Tr = trust establishment; AL = operational assurance and authorization-lifecycle behavior; DLT = functional analysis of DLT/SC roles. E = explicit analytical focus; P = partial or indirect coverage; – = not a primary organizing dimension. Search-window descriptions are based on the wording reported by the reviewed articles and are normalized for consistency where qualitative periods are used; “Not stated” indicates that no search window was reported. Where a qualitative period such as “Recent years” or “Past decades” is shown, the reviewed article did not specify exact calendar-year boundaries. The entries indicate analytical emphasis rather than whether an individual topic is mentioned anywhere in a reviewed article.

**Table 2:** Summary of the seven tenets of Zero Trust Architecture based on NIST SP 800-207 [20].

Tenet	Summary
T1	All data sources and computing services are treated as resources, including sensors and personal devices used for enterprise purposes.
T2	Communications are secured regardless of network location.
T3	Access to individual enterprise resources is granted on a per-session basis, with bounded duration and possible renewal or reauthorization.
T4	Access decisions are based on dynamic policy using identity, asset state, behavioral, environmental, and other contextual attributes.
T5	The integrity and security posture of owned and associated assets are continuously monitored and assessed.
T6	Authentication and authorization are dynamically and strictly enforced, with possible reauthentication, reauthorization, and revocation during transactions.
T7	Information about assets, networks, communications, and access activity is collected and used to improve security posture and policy enforcement.

**Table 3:** Logical components of the Zero Trust Architecture reference model relevant to access control.

Component	Role
Policy Decision Point (PDP)	Makes authorization decisions for access to enterprise resources. In the ZTA reference architecture, the PDP comprises the Policy Engine (PE) and Policy Administrator (PA).
Policy Engine (PE)	Evaluates access requests using enterprise policies and contextual information obtained from sources such as identity-management, asset-state, and threat-intelligence systems.
Policy Administrator (PA)	Establishes or manages the communication path between a subject and an enterprise resource according to the PE’s decision. It may generate session-specific credentials or configuration and instruct the PEP to establish or terminate a session.
Policy Enforcement Point (PEP)	Controls and monitors access between subjects and enterprise resources and enforces authorization decisions received from the PDP.
Subject	A person, application, or service that requests access to an enterprise resource through a system.
System	A device or computing component through which the subject requests access, such as a computer, smartphone, server, gateway, or IoT device.
Enterprise resource	Data, applications, services, systems, or devices protected by the enterprise.

**Table 4:** Information sources in the Zero Trust Architecture reference model relevant to authorization analysis.

Information Source	Role in Authorization
Continuous Diagnostics and Mitigation (CDM) system	Provides information about the security posture and configuration of assets, including software versions and other state information used in contextual access decisions.
Threat intelligence feeds	Supply internal and external information about threats, vulnerabilities, malware, and observed attacks that may influence policy evaluation.
Activity logs	Record asset, network, and resource-access events and provide operational feedback for monitoring, accountability, and policy refinement.
Data access policies	Define authorization rules using subject, resource, action, device, and environmental attributes and provide the policy basis for access decisions.
Public Key Infrastructure (PKI)	Issues and manages certificates and credentials used to authenticate subjects, systems, services, and resources and to establish trust.
Identity management system	Maintains identities, credentials, roles, attributes, and assigned assets used as inputs to authorization decisions.
Security Information and Event Management (SIEM) system	Collects and analyzes security-related events to support policy refinement, risk assessment, and potential reauthorization or revocation actions.

**Table 5:** Comparison between XACML and OAuth 2.0.

Aspect	XACML	OAuth 2.0
Purpose	Policy-based authorization, commonly used for ABAC	Delegated and limited access to protected resources
Authorization mechanism	Policy evaluation using PDP/PEP and related components	Token-based authorization using scopes and authorization grants
Typical use	Enterprise systems, cloud services, and distributed policy enforcement	Web services, APIs, mobile applications, and service-to-service access
Authentication	Not defined; identity and attributes are provided by external systems	Not defined; commonly combined with OIDC when authentication is required
Decision and enforcement	Authorization decisions and enforcement are logically separated through PDP and PEP	Authorization is granted by an authorization server and enforced by a resource server
Standard	OASIS XACML 3.0	IETF RFC 6749 and RFC 6750 [3,47]

**Table 6:** Illustrative mapping of representative access control and identity models to criteria C1–C5.

Period	Model	C1	C2	C3	C4 (Typical)	C5	Representative Examples
1970s–1980s	MAC	Admin	Rule-based	Centralized	Central PDP/Local PEP	Local administrative trust	Bell–LaPadula, Biba, LOMAC, DTE
1970s–1980s	DAC	Owner	Rule-based	Centralized	Central PDP/Local PEP	Local administrative trust	UNIX permissions
1990s–2000s	RBAC	Admin	RBAC	Centralized	Central PDP/Local PEP	Local administrative trust	NIST RBAC
1990s–2000s	ABAC	Admin	ABAC	Centralized	Central PDP/Local PEP	Deployment-dependent	XACML, OPA
2000s onward	CAP-BAC	Varies	Varies	Varies	Local or on-resource verification is common	Deployment-dependent	Capability tokens, Macaroons
2000s onward	UCON	Varies	Varies	Varies	Deployment-dependent; continuous authorization	Deployment-dependent	UCONABC; ongoing authorization, obligations, conditions, and attribute mutability [46]
2010s onward	ReBAC	Varies	ReBAC	Varies	Deployment-dependent	Deployment-dependent	Relationship graphs, Fong’s ReBAC model
2010s onward	SSI	Varies	Varies	User-held	Deployment-dependent	SSI (DID/VC)	DIDs, VCs, Sovrin

*Note:* C1–C5 denote Policy Governance, Decision Basis, Identity and Attribute Management, PDP–PEP Architecture, and Trust Establishment, respectively. The criteria are defined in Section 4 and Appendix C. The periods are approximate and indicate historical emphasis rather than exact publication dates. The entries illustrate general tendencies rather than intrinsic properties or corpus coding results. “Varies” indicates implementation-dependent characteristics. C6 is omitted because operational-assurance and auditability mechanisms are not consistently defined for these generic model families.

**Table 7:** Standards traceability and coding operationalization of the C1–C6 classification criteria.

ID	Criterion	Analytical Rationale/Core Question	Conceptual/Standards Basis	Coding Operationalization
C1	Policy Governance	Separates policy authority from technical placement: who controls policy definition, update, delegation, or revocation?	XACML PAP [1]; OAuth 2.0 delegated access [3]; NGAC/Policy Machine [44]; IETF NMOP policy sharing [28]	Primary + optional secondary: Admin, Owner, Consortium, Hybrid, or Delegated.
C2	Decision Basis	Separates decision semantics from credential and placement choices: what primarily determines the authorization decision?	NIST RBAC [35]; NIST ABAC [36]; ReBAC [40]; XACML rule-based authorization [1]	Primary + optional secondary: RBAC, ABAC, ReBAC, or Rule-based. Rule-based subtypes are recorded separately.
C3	Identity and Attribute Management	Separates identity/attribute administration from decision placement: where and by whom are authorization-relevant identities and attributes managed?	LDAP [48]; federated identity/OIDC [4]; W3C DIDs and VCs [52,53]; holder-mediated presentation [5]	Primary + optional secondary: Centralized, Federated, Distributed, or User-held.
C4	PDP–PEP Architecture	Captures logical decision/enforcement placement: where are PDP and PEP functions located and separated?	XACML PDP–PEP architecture [1]; NIST ZTA logical components [20]; capability-based local verification [42]	Primary placement: Central PDP/Local PEP, Distributed PDP/Local PEP, Hierarchical PDP/Local PEP, or On-resource. Subtypes are recorded separately.
C5	Trust Establishment	Separates trust basis from governance and placement: what primarily establishes authorization-relevant trust?	NIST ZTA trust inputs [20]; federated identity/OIDC [4]; W3C DIDs and VCs [52,53]; permissioned/ledger trust [55]	Primary + optional secondary: Local administrative, PKI, Federation, SSI, Consortium, or Ledger-anchored integrity.
C6	Operational Assurance and Auditability	Captures assurance beyond C1–C5: what mechanisms provide logging, auditability, tamper evidence, or policy-state change control?	NIST audit/accountability guidance [23]; ISO/IEC 27001 [24]; NIST ZTA operational feedback [20]; IETF NMOP policy lifecycle [28]	Multi-label: Central logging, Tamper-evident log, Verifiable audit, and Policy change control; “– (Not specified)” if none is reported.

**Table 8:** Use of selected ZTA tenets as authorization-oriented analytical indicators.

Tenet	Used?	Rationale and Scope
T1	No	Defines the scope of resources; relevant to system modeling but not used as an authorization-semantic indicator.
T2	No	Concerns the protection of communications regardless of network location rather than authorization decisions or enforcement semantics.
T3	Yes	Evaluates whether authorization is explicitly bounded by a session, validity interval, expiration, lease, or limited-use condition; renewal or reauthorization may also be described.
T4	Yes	Evaluates whether authorization decisions use dynamic policy inputs, contextual attributes, or observable asset and environmental state.
T5	No	Concerns asset-integrity and security-posture monitoring; resulting signals may inform T4 or trigger T6-related actions but are not evaluated independently.
T6	Yes	Evaluates the authorization-related aspect of dynamic enforcement, including request-time or current-state validation, reauthorization, revocation or status checking, and policy-triggered re-evaluation.
T7	No	Concerns telemetry collection and security analytics; resulting information may support policy refinement or lifecycle actions but is not evaluated independently.

**Table 9:** Publisher/platform distribution of the final mapping corpus.

Publisher/Platform	Studies	Percentage
IEEE	33	36.7%
Springer	14	15.6%
ACM	5	5.6%
MDPI	14	15.6%
Preprint platforms	3	3.3%
Elsevier	9	10.0%
Other	12	13.3%
Total	90	100.0%

Note: Percentages may not sum to 100.0% because of rounding. For jointly sponsored venues, studies were classified according to the publisher of the proceedings.

**Table 10:** Mapping of selected studies by application area and classification criteria C1–C3, C5, and C6.

Study	Area	C1	C2	C3	C5	C6
Gonzalez-Gil et al. [57] (2020)	IoT	Admin	ABAC	Centralized	Local administrative trust	PC
Siris et al. [58] (2020)	IoT	Owner	Rule-based	Federated	Ledger-anchored integrity	TE, VA
Bakir et al. [59] (2021)	IoT	Owner	Rule-based	Centralized	Local administrative trust	VA, PC
Li et al. [60] (2021)	IoT	Owner	RBAC	Distributed	Ledger-anchored integrity	TE, PC
Mishra et al. [61] (2021)	IoT	Hybrid	Rule-based	Distributed	Ledger-anchored integrity	TE, PC
Ouaddah and Bellaj [62] (2021)	IoT	Owner	ABAC	User-held	Ledger-anchored integrity	TE, PC
Putra et al. [63] (2021)	IoT	Owner	ABAC	Distributed	Ledger-anchored integrity	TE, PC
Ren et al. [64] (2021)	IoT	Admin	ABAC	Federated	Consortium trust	TE, VA, PC
Riabi et al. [65] (2021)	IoT	Owner	Rule-based	Distributed	Ledger-anchored integrity	TE, PC
Sylla et al. [66] (2021)	IoT	Owner	Rule-based	Distributed	Ledger-anchored integrity	TE, PC
Zaidi et al. [67] (2021)	IoT	Owner	ABAC	Distributed	Ledger-anchored integrity	TE, PC
Zhang et al. [68] (2021)	IoT	Admin	ABAC	Distributed	Ledger-anchored integrity	TE, PC
Zhang et al. [69] (2021)	IoT	Owner	ABAC	Centralized	Local administrative trust	TE, PC
Zhong et al. [70] (2021)	IoT	Owner	Rule-based	Distributed	Consortium trust	TE
Abdi et al. [71] (2022)	IoT	Admin	ABAC	Distributed	Consortium trust	TE, PC
Chen et al. [72] (2022)	IoT	Owner	ABAC	Distributed	Consortium trust	TE, VA, PC
Fedrecheski et al. [73] (2022)	IoT	Owner	ABAC	Distributed	Local administrative trust	–
Fotiou et al. [74] (2022)	IoT	Delegated	Rule-based	User-held	SSI (DID/VC)	–
Hameed et al. [75] (2022)	IoT	Admin	ABAC	Centralized	Ledger-anchored integrity	TE, VA, PC
Han et al. [76] (2022)	IoT	Owner	ABAC	Centralized	Consortium trust	TE
Saidi et al. [77] (2022)	IoT	Owner	RBAC	User-held	SSI (DID/VC)	TE, PC
Savvaidis et al. [78] (2022)	IoT	Admin	ABAC	Distributed	Consortium trust	TE, VA, PC
Shakarami et al. [79] (2022)	IoT	Delegated	RBAC	Distributed	Ledger-anchored integrity	TE, VA, PC
Shammar et al. [80] (2022)	IoT	Admin	ABAC	Federated	Consortium trust	TE, PC
Wang et al. [81] (2022)	IoT	Owner	ABAC	Federated	Local administrative trust	TE, VA, PC
Yang et al. [82] (2022)	IoT	Admin	RBAC	Centralized	Ledger-anchored integrity	CL
Zhai et al. [83] (2022)	IoT	Admin	ABAC	Distributed	Ledger-anchored integrity	TE, PC
Fotiou et al. [84] (2023)	IoT	Owner	Rule-based	User-held	SSI (DID/VC)	–
Han et al. [85] (2023)	IoT	Owner	ABAC	Centralized	Local administrative trust	TE, VA
Hasan et al. [86] (2023)	IoT	Owner	Rule-based	Distributed	Ledger-anchored integrity	TE, PC
Hu et al. [87] (2023)	IoT	Owner	Rule-based	Centralized	Ledger-anchored integrity	TE, VA
Jiang et al. [88] (2023)	IoT	Admin	ABAC	Distributed	Ledger-anchored integrity	PC
Malamas et al. [89] (2023)	IoT	Consortium	ABAC	Federated	Consortium trust	TE, VA, PC
Mishra et al. [90] (2023)	IoT	Hybrid	Rule-based	Federated	Consortium trust	TE, PC
Pateritsas and Petrakis [91] (2023)	IoT	Admin	RBAC	User-held	SSI (DID/VC)	TE
Pham et al. [92] (2023)	IoT	Owner	ABAC	User-held	Local administrative trust	TE, PC
Pittaras and Polyzos [93] (2023)	IoT	Owner	Rule-based	Distributed	Ledger-anchored integrity	TE, PC
Polychronaki et al. [94] (2023)	IoT	Owner	ReBAC	User-held	Ledger-anchored integrity	TE, PC
Salin [95] (2023)	IoT	Owner	RBAC	Distributed	Local administrative trust	PC
Wang et al. [96] (2023)	IoT	Owner	Rule-based	Distributed	Ledger-anchored integrity	TE
Yan et al. [97] (2023)	IoT	Admin	ABAC	Distributed	Ledger-anchored integrity	TE, VA
Yang et al. [98] (2023)	IoT	Owner	Rule-based	Distributed	Ledger-anchored integrity	TE, PC
Abid et al. [99] (2024)	IoT	Owner	RBAC	Distributed	Ledger-anchored integrity	TE
Huang et al. [100] (2024)	IoT	Hybrid	Rule-based	Distributed	Consortium trust	TE, PC
Huang et al. [101] (2024)	IoT	Hybrid	Rule-based	Distributed	Consortium trust	TE, PC
Khan et al. [102] (2024)	IoT	Owner	RBAC	Distributed	Ledger-anchored integrity	TE
Rajesh and Prabha [103] (2024)	IoT	Owner	ABAC	Distributed	Ledger-anchored integrity	TE
Rashid et al. [104] (2024)	IoT	Delegated	Rule-based	Distributed	Ledger-anchored integrity	TE, VA, PC
Wang et al. [105] (2024)	IoT	Owner	ABAC	Distributed	Consortium trust	TE
Yang et al. [106] (2024)	IoT	Owner	ABAC	Distributed	Ledger-anchored integrity	TE, VA
Zhang et al. [107] (2024)	IoT	Owner	Rule-based	Distributed	Ledger-anchored integrity	TE
Chen et al. [108] (2025)	IoT	Owner	ABAC	Distributed	Local administrative trust	PC
Fang et al. [109] (2025)	IoT	Consortium	Rule-based	Distributed	Consortium trust	TE
Fedrecheski et al. [110] (2025)	IoT	Owner	ABAC	User-held	SSI (DID/VC)	–
Kavitha and Gayathri Devi [111] (2025)	IoT	Hybrid	ABAC	Federated	Ledger-anchored integrity	TE, PC
Ravalika et al. [112] (2025)	IoT	Admin	Rule-based	Distributed	Ledger-anchored integrity	TE, PC
Stodt et al. [113] (2025)	IoT	Consortium	ABAC	Federated	Consortium trust	TE, VA, PC
Waheed et al. [114] (2025)	IoT	Admin	ABAC	Distributed	PKI-based trust	TE, VA, PC
Akram et al. [115] (2026)	IoT	Admin	Rule-based	User-held	SSI (DID/VC)	–
Díaz-Sánchez et al. [116] (2026)	IoT	Delegated	Rule-based	Federated	Federation trust	–
Goswami and Kadao [117] (2026)	IoT	Admin	ABAC	Distributed	Ledger-anchored integrity	TE
Han et al. [118] (2026)	IoT	Delegated	Rule-based	Federated	Ledger-anchored integrity	TE
Kang et al. [119] (2026)	IoT	Owner	Rule-based	Distributed	Consortium trust	TE, VA, PC
Kumar et al. [120] (2026)	IoT	Admin	Rule-based	Centralized	Ledger-anchored integrity	TE
Mailapalli and Sheela [121] (2026)	IoT	Consortium	ABAC	Distributed	Ledger-anchored integrity	TE, VA, PC
Upadhyay et al. [122] (2026)	IoT	Admin	RBAC	Federated	Ledger-anchored integrity	TE, VA, PC
Zhang et al. [123] (2026)	IoT	Admin	ABAC	Distributed	Ledger-anchored integrity	TE
Kim et al. [124] (2020)	Edge	Admin	Rule-based	Federated	Federation trust	–
Algarni et al. [125] (2021)	Edge	Admin	Rule-based	Distributed	Ledger-anchored integrity	TE, PC
Dougherty et al. [126] (2021)	Edge	Hybrid	Rule-based	Federated	Federation trust	–
Zhu et al. [127] (2021)	Edge	Admin	RBAC	Distributed	Ledger-anchored integrity	TE, PC
Frimpong et al. [128] (2022)	Edge	Hybrid	ABAC	Centralized	Local administrative trust	PC
Zhu et al. [129] (2022)	Edge	Hybrid	ABAC	Distributed	Ledger-anchored integrity	TE, VA, PC
Alzahrani et al. [130] (2023)	Edge	Delegated	Rule-based	User-held	PKI-based trust	–
Pathak et al. [131] (2023)	Edge	Admin	ABAC	Centralized	PKI-based trust	TE, PC
Song et al. [132] (2023)	Edge	Owner	ABAC	Centralized	Local administrative trust	VA, PC
Zhou et al. [133] (2023)	Edge	Admin	Rule-based	Federated	Federation trust	VA
Andalib et al. [134] (2024)	Edge	Admin	ABAC	Centralized	Ledger-anchored integrity	TE, PC
Liu et al. [135] (2024)	Edge	Admin	Rule-based	Centralized	Local administrative trust	–
Ellouze et al. [136] (2025)	Edge	Owner	Rule-based	Distributed	Ledger-anchored integrity	TE, PC
Sheetal et al. [137] (2025)	Edge	Admin	Rule-based	Distributed	Ledger-anchored integrity	TE, VA
Chen et al. [138] (2026)	Edge	Admin	Rule-based	Federated	Local administrative trust	VA
Ullah et al. [139] (2026)	Edge	Admin	ABAC	Distributed	Ledger-anchored integrity	TE, PC
Shafagh et al. [140] (2020)	WSN	Owner	Rule-based	User-held	Ledger-anchored integrity	TE, VA, PC
Trabelsi et al. [141] (2023)	WSN	Owner	Rule-based	Centralized	Ledger-anchored integrity	TE, PC
dos Santos et al. [142] (2026)	WSN	Owner	ABAC	Federated	Consortium trust	TE, VA, PC
Jyosthna et al. [143] (2026)	WSN	Admin	Rule-based	Centralized	Local administrative trust	–
Kchaou et al. [144] (2020)	V2X	Admin	ABAC	Distributed	Ledger-anchored integrity	TE, PC
Lu et al. [145] (2026)	V2X	Owner	ABAC	Distributed	Federation trust	–
Zhi et al. [146] (2026)	V2X	Admin	ABAC	Centralized	Local administrative trust	PC

Note: C6 abbreviations: CL = Central logging, TE = Tamper-evident log, VA = Verifiable audit, and PC = Policy change control.

**Table 11:** Application areas, PDP–PEP architectural placement patterns (C4), and C4 subtypes of the selected studies. “Token-based” denotes general token use, whereas “CAP-BAC” denotes an independently verifiable capability-based authorization grant; subtype labels are not mutually exclusive.

Study	Area	C4 Pattern	C4 Subtype
Gonzalez-Gil et al. [57] (2020)	IoT	Central PDP/Local PEP	CAP-BAC; Token-based
Siris et al. [58] (2020)	IoT	Distributed PDP/Local PEP	Token-based; CAP-BAC; On-chain PDP; Multi-authority
Bakir et al. [59] (2021)	IoT	Central PDP/Local PEP	Token-based; CAP-BAC
Li et al. [60] (2021)	IoT	Distributed PDP/Local PEP	On-chain PDP; Aggregated-at-gateway
Mishra et al. [61] (2021)	IoT	Distributed PDP/Local PEP	Token-based; CAP-BAC; On-chain PDP
Ouaddah and Bellaj [62] (2021)	IoT	Distributed PDP/Local PEP	Token-based; CAP-BAC; On-chain PDP
Putra et al. [63] (2021)	IoT	Distributed PDP/Local PEP	On-chain PDP; Token-based; Multi-authority
Ren et al. [64] (2021)	IoT	Distributed PDP/Local PEP	Token-based; Crypto-enforced; CAP-BAC; On-chain PDP; Multi-authority
Riabi et al. [65] (2021)	IoT	Distributed PDP/Local PEP	Token-based; CAP-BAC; On-chain PDP
Sylla et al. [66] (2021)	IoT	Hierarchical PDP/Local PEP	Token-based; CAP-BAC; On-chain PDP
Zaidi et al. [67] (2021)	IoT	Distributed PDP/Local PEP	On-chain PDP; Crypto-enforced
Zhang et al. [68] (2021)	IoT	Distributed PDP/Local PEP	On-chain PDP
Zhang et al. [69] (2021)	IoT	Distributed PDP/Local PEP	Token-based; Crypto-enforced; CAP-BAC; Aggregated-at-gateway
Zhong et al. [70] (2021)	IoT	Central PDP/Local PEP	Token-based; CAP-BAC
Abdi et al. [71] (2022)	IoT	Hierarchical PDP/Local PEP	On-chain PDP; Aggregated-at-gateway
Chen et al. [72] (2022)	IoT	Distributed PDP/Local PEP	On-chain PDP
Fedrecheski et al. [73] (2022)	IoT	On-resource decision + enforcement	–
Fotiou et al. [74] (2022)	IoT	Central PDP/Local PEP	Token-based; CAP-BAC; Multi-authority; SSI
Hameed et al. [75] (2022)	IoT	Distributed PDP/Local PEP	Token-based; CAP-BAC; On-chain PDP
Han et al. [76] (2022)	IoT	Distributed PDP/Local PEP	On-chain PDP
Saidi et al. [77] (2022)	IoT	Distributed PDP/Local PEP	On-chain PDP; Token-based; SSI
Savvaidis et al. [78] (2022)	IoT	Distributed PDP/Local PEP	On-chain PDP
Shakarami et al. [79] (2022)	IoT	Hierarchical PDP/Local PEP	On-chain PDP; Aggregated-at-gateway
Shammar et al. [80] (2022)	IoT	Distributed PDP/Local PEP	On-chain PDP
Wang et al. [81] (2022)	IoT	Distributed PDP/Local PEP	On-chain PDP; Multi-authority
Yang et al. [82] (2022)	IoT	Distributed PDP/Local PEP	On-chain PDP; Token-based; Aggregated-at-gateway
Zhai et al. [83] (2022)	IoT	Distributed PDP/Local PEP	On-chain PDP; Multi-authority
Fotiou et al. [84] (2023)	IoT	Central PDP/Local PEP	Token-based; CAP-BAC; Aggregated-at-gateway; Multi-authority; SSI
Han et al. [85] (2023)	IoT	Distributed PDP/Local PEP	On-chain PDP; Crypto-enforced
Hasan et al. [86] (2023)	IoT	Distributed PDP/Local PEP	On-chain PDP
Hu et al. [87] (2023)	IoT	Distributed PDP/Local PEP	Token-based; CAP-BAC; On-chain PDP
Jiang et al. [88] (2023)	IoT	Distributed PDP/Local PEP	On-chain PDP; Aggregated-at-gateway
Malamas et al. [89] (2023)	IoT	Hierarchical PDP/Local PEP	Crypto-enforced; Multi-authority; On-chain PDP
Mishra et al. [90] (2023)	IoT	Distributed PDP/Local PEP	On-chain PDP; Aggregated-at-gateway; Multi-authority
Pateritsas and Petrakis [91] (2023)	IoT	On-resource decision + enforcement	Token-based; SSI
Pham et al. [92] (2023)	IoT	Distributed PDP/Local PEP	On-chain PDP; Aggregated-at-gateway; Multi-authority; SSI
Pittaras and Polyzos [93] (2023)	IoT	Distributed PDP/Local PEP	On-chain PDP; Token-based
Polychronaki et al. [94] (2023)	IoT	Distributed PDP/Local PEP	On-chain PDP; SSI
Salin [95] (2023)	IoT	On-resource decision + enforcement	–
Wang et al. [96] (2023)	IoT	Distributed PDP/Local PEP	Token-based; CAP-BAC; On-chain PDP
Yan et al. [97] (2023)	IoT	Distributed PDP/Local PEP	On-chain PDP
Yang et al. [98] (2023)	IoT	Distributed PDP/Local PEP	Token-based; CAP-BAC; On-chain PDP
Abid et al. [99] (2024)	IoT	Distributed PDP/Local PEP	On-chain PDP; Aggregated-at-gateway
Huang et al. [100] (2024)	IoT	Distributed PDP/Local PEP	Token-based; CAP-BAC; Aggregated-at-gateway
Huang et al. [101] (2024)	IoT	Distributed PDP/Local PEP	Token-based; CAP-BAC; Aggregated-at-gateway
Khan et al. [102] (2024)	IoT	Distributed PDP/Local PEP	On-chain PDP
Rajesh and Prabha [103] (2024)	IoT	Distributed PDP/Local PEP	On-chain PDP
Rashid et al. [104] (2024)	IoT	Distributed PDP/Local PEP	On-chain PDP
Wang et al. [105] (2024)	IoT	Distributed PDP/Local PEP	Crypto-enforced; Multi-authority; On-chain PDP
Yang et al. [106] (2024)	IoT	On-resource decision + enforcement	Crypto-enforced; Aggregated-at-gateway
Zhang et al. [107] (2024)	IoT	Distributed PDP/Local PEP	Token-based; On-chain PDP
Chen et al. [108] (2025)	IoT	Central PDP/Local PEP	Token-based
Fang et al. [109] (2025)	IoT	Distributed PDP/Local PEP	On-chain PDP; Token-based; Multi-authority
Fedrecheski et al. [110] (2025)	IoT	On-resource decision + enforcement	Token-based; CAP-BAC; SSI
Kavitha and Gayathri Devi [111] (2025)	IoT	Hierarchical PDP/Local PEP	Crypto-enforced; On-chain PDP; Multi-authority
Ravalika et al. [112] (2025)	IoT	On-resource decision + enforcement	–
Stodt et al. [113] (2025)	IoT	Distributed PDP/Local PEP	Multi-authority
Waheed et al. [114] (2025)	IoT	Central PDP/Local PEP	On-chain PDP
Akram et al. [115] (2026)	IoT	Central PDP/Local PEP	Token-based; CAP-BAC; Aggregated-at-gateway; Multi-authority; SSI
Díaz-Sánchez et al. [116] (2026)	IoT	Hierarchical PDP/Local PEP	Token-based; CAP-BAC; Multi-authority
Goswami and Kadao [117] (2026)	IoT	Distributed PDP/Local PEP	On-chain PDP
Han et al. [118] (2026)	IoT	Distributed PDP/Local PEP	Multi-authority
Kang et al. [119] (2026)	IoT	Distributed PDP/Local PEP	On-chain PDP; Aggregated-at-gateway
Kumar et al. [120] (2026)	IoT	Distributed PDP/Local PEP	On-chain PDP; Aggregated-at-gateway
Mailapalli and Sheela [121] (2026)	IoT	Distributed PDP/Local PEP	On-chain PDP; Aggregated-at-gateway
Upadhyay et al. [122] (2026)	IoT	Hierarchical PDP/Local PEP	On-chain PDP; Multi-authority; Aggregated-at-gateway
Zhang et al. [123] (2026)	IoT	Distributed PDP/Local PEP	On-chain PDP
Kim et al. [124] (2020)	Edge	Distributed PDP/Local PEP	–
Algarni et al. [125] (2021)	Edge	Hierarchical PDP/Local PEP	Aggregated-at-gateway
Dougherty et al. [126] (2021)	Edge	On-resource decision + enforcement	Token-based; CAP-BAC; Crypto-enforced; Multi-authority
Zhu et al. [127] (2021)	Edge	Distributed PDP/Local PEP	On-chain PDP; Aggregated-at-gateway
Frimpong et al. [128] (2022)	Edge	Distributed PDP/Local PEP	Aggregated-at-gateway
Zhu et al. [129] (2022)	Edge	Distributed PDP/Local PEP	Token-based; On-chain PDP; Aggregated-at-gateway
Alzahrani et al. [130] (2023)	Edge	Central PDP/Local PEP	Token-based; CAP-BAC; Aggregated-at-gateway; Multi-authority; SSI
Pathak et al. [131] (2023)	Edge	Distributed PDP/Local PEP	On-chain PDP
Song et al. [132] (2023)	Edge	On-resource decision + enforcement	Crypto-enforced
Zhou et al. [133] (2023)	Edge	On-resource decision + enforcement	Crypto-enforced; Multi-authority
Andalib et al. [134] (2024)	Edge	Hierarchical PDP/Local PEP	On-chain PDP; Aggregated-at-gateway
Liu et al. [135] (2024)	Edge	Central PDP/Local PEP	Token-based; Crypto-enforced; CAP-BAC
Ellouze et al. [136] (2025)	Edge	Distributed PDP/Local PEP	On-chain PDP; Crypto-enforced
Sheetal et al. [137] (2025)	Edge	Hierarchical PDP/Local PEP	On-chain PDP; Aggregated-at-gateway
Chen et al. [138] (2026)	Edge	On-resource decision + enforcement	Token-based; Crypto-enforced; CAP-BAC; Multi-authority
Ullah et al. [139] (2026)	Edge	Distributed PDP/Local PEP	Token-based; Aggregated-at-gateway
Shafagh et al. [140] (2020)	WSN	Distributed PDP/Local PEP	Token-based; Crypto-enforced; CAP-BAC
Trabelsi et al. [141] (2023)	WSN	Distributed PDP/Local PEP	On-chain PDP; Aggregated-at-gateway
dos Santos et al. [142] (2026)	WSN	Central PDP/Local PEP	On-chain PDP; Aggregated-at-gateway
Jyosthna et al. [143] (2026)	WSN	Central PDP/Local PEP	–
Kchaou et al. [144] (2020)	V2X	Distributed PDP/Local PEP	Token-based
Lu et al. [145] (2026)	V2X	Distributed PDP/Local PEP	Crypto-enforced; Multi-authority
Zhi et al. [146] (2026)	V2X	Distributed PDP/Local PEP	Token-based; Crypto-enforced; CAP-BAC

**Table 12:** Correspondence of selected studies with authorization-relevant aspects of the selected ZTA tenets and reported DLT/SC usage by application area.

Study	Area	T3	T4	T6	DLT/SC	DLT/SC Usage Tags
Gonzalez-Gil et al. [57] (2020)	IoT	Yes	No	Partial	No	–
Siris et al. [58] (2020)	IoT	Yes	Partial	Partial	Yes	Policy-eval; Policy-store; Trust-anchor; Audit-log; Coordination
Bakir et al. [59] (2021)	IoT	Yes	Yes	Yes	No	–
Li et al. [60] (2021)	IoT	No	No	Yes	Yes	Policy-eval; Policy-store; Trust-anchor; Audit-log
Mishra et al. [61] (2021)	IoT	No	No	No	Yes	Policy-eval; Policy-store; Trust-anchor; Audit-log
Ouaddah and Bellaj [62] (2021)	IoT	Yes	Yes	Yes	Yes	Policy-eval; Trust-anchor; Audit-log; Policy-store; Governance; Coordination
Putra et al. [63] (2021)	IoT	Yes	Yes	Yes	Yes	Policy-eval; Policy-store; Attribute-registry; Trust-anchor; Audit-log; Coordination
Ren et al. [64] (2021)	IoT	Yes	Yes	Yes	Yes	Policy-eval; Trust-anchor; Audit-log; Governance
Riabi et al. [65] (2021)	IoT	Yes	No	Partial	Yes	Policy-eval; Policy-store; Trust-anchor; Audit-log; Governance
Sylla et al. [66] (2021)	IoT	Yes	Yes	Yes	Yes	Policy-eval; Trust-anchor; Audit-log; Policy-store; Governance; Coordination
Zaidi et al. [67] (2021)	IoT	Yes	No	Partial	Yes	Policy-eval; Policy-store; Attribute-registry; Trust-anchor; Audit-log
Zhang et al. [68] (2021)	IoT	Yes	Yes	Yes	Yes	Policy-eval; Policy-store; Attribute-registry; Trust-anchor; Audit-log
Zhang et al. [69] (2021)	IoT	No	No	Yes	Yes	Policy-store; Trust-anchor; Audit-log
Zhong et al. [70] (2021)	IoT	Yes	No	Partial	Yes	Trust-anchor; Audit-log
Abdi et al. [71] (2022)	IoT	Yes	Yes	Partial	Yes	Policy-eval; Trust-anchor; Audit-log; Attribute-registry; Policy-store; Governance; Coordination
Chen et al. [72] (2022)	IoT	No	Yes	Yes	Yes	Policy-eval; Policy-store; Trust-anchor; Audit-log; Governance
Fedrecheski et al. [73] (2022)	IoT	No	Yes	Partial	No	–
Fotiou et al. [74] (2022)	IoT	Yes	No	Yes	No	–
Hameed et al. [75] (2022)	IoT	Partial	Yes	Partial	Yes	Policy-eval; Policy-store; Attribute-registry; Trust-anchor; Audit-log
Han et al. [76] (2022)	IoT	Yes	No	Yes	Yes	Policy-eval; Policy-store; Trust-anchor; Audit-log; Attribute-registry
Saidi et al. [77] (2022)	IoT	No	Yes	Partial	Yes	Policy-eval; Trust-anchor; Audit-log; Attribute-registry; Policy-store
Savvaidis et al. [78] (2022)	IoT	No	Partial	Yes	Yes	Policy-eval; Policy-store; Attribute-registry; Trust-anchor; Audit-log
Shakarami et al. [79] (2022)	IoT	Yes	Yes	Yes	Yes	Policy-eval; Trust-anchor; Audit-log; Policy-store; Governance; Coordination
Shammar et al. [80] (2022)	IoT	Yes	Yes	Yes	Yes	Policy-eval; Policy-store; Trust-anchor; Audit-log
Wang et al. [81] (2022)	IoT	Yes	Yes	Yes	Yes	Policy-eval; Policy-store; Trust-anchor; Audit-log
Yang et al. [82] (2022)	IoT	Yes	Yes	Partial	Yes	Policy-eval; Policy-store; Trust-anchor
Zhai et al. [83] (2022)	IoT	No	Yes	Yes	Yes	Policy-eval; Trust-anchor; Audit-log; Attribute-registry; Policy-store
Fotiou et al. [84] (2023)	IoT	Yes	No	Yes	No	–
Han et al. [85] (2023)	IoT	No	Yes	Yes	Yes	Policy-eval; Trust-anchor; Audit-log; Coordination
Hasan et al. [86] (2023)	IoT	Yes	Yes	Yes	Yes	Policy-eval; Trust-anchor; Audit-log; Attribute-registry; Policy-store; Governance
Hu et al. [87] (2023)	IoT	Yes	No	Yes	Yes	Policy-eval; Trust-anchor; Audit-log; Policy-store
Jiang et al. [88] (2023)	IoT	No	Yes	Yes	Yes	Policy-eval; Trust-anchor; Attribute-registry; Policy-store; Governance; Coordination
Malamas et al. [89] (2023)	IoT	Yes	Yes	Yes	Yes	Policy-eval; Policy-store; Attribute-registry; Trust-anchor; Audit-log; Governance; Coordination
Mishra et al. [90] (2023)	IoT	No	Yes	Yes	Yes	Policy-eval; Policy-store; Attribute-registry; Trust-anchor; Audit-log; Governance
Pateritsas and Petrakis [91] (2023)	IoT	Yes	No	Partial	Yes	Attribute-registry; Trust-anchor; Audit-log
Pham et al. [92] (2023)	IoT	Yes	Yes	Yes	Yes	Policy-eval; Policy-store; Attribute-registry; Trust-anchor; Audit-log
Pittaras and Polyzos [93] (2023)	IoT	No	No	Yes	Yes	Policy-eval; Policy-store; Trust-anchor; Audit-log
Polychronaki et al. [94] (2023)	IoT	No	No	Yes	Yes	Policy-eval; Policy-store; Attribute-registry; Trust-anchor; Audit-log
Salin [95] (2023)	IoT	No	No	Yes	No	–
Wang et al. [96] (2023)	IoT	No	No	Yes	Yes	Policy-eval; Trust-anchor; Audit-log; Attribute-registry; Policy-store; Governance
Yan et al. [97] (2023)	IoT	Partial	Yes	Yes	Yes	Policy-eval; Policy-store; Attribute-registry; Trust-anchor; Audit-log
Yang et al. [98] (2023)	IoT	Yes	Yes	Yes	Yes	Policy-eval; Policy-store; Attribute-registry; Trust-anchor; Audit-log
Abid et al. [99] (2024)	IoT	Yes	Yes	Yes	Yes	Policy-eval; Trust-anchor; Audit-log; Policy-store; Coordination
Huang et al. [100] (2024)	IoT	Yes	Partial	Partial	Yes	Policy-store; Attribute-registry; Trust-anchor; Audit-log
Huang et al. [101] (2024)	IoT	Yes	Partial	Yes	Yes	Policy-eval; Policy-store; Attribute-registry; Trust-anchor; Audit-log
Khan et al. [102] (2024)	IoT	No	No	Partial	Yes	Policy-eval; Policy-store; Trust-anchor; Audit-log
Rajesh and Prabha [103] (2024)	IoT	No	Yes	Yes	Yes	Policy-eval; Policy-store; Attribute-registry; Trust-anchor; Audit-log
Rashid et al. [104] (2024)	IoT	No	No	Yes	Yes	Policy-eval; Trust-anchor; Audit-log; Attribute-registry; Policy-store; Governance; Coordination
Wang et al. [105] (2024)	IoT	No	No	Partial	Yes	Policy-eval; Policy-store; Attribute-registry; Trust-anchor; Audit-log; Coordination
Yang et al. [106] (2024)	IoT	No	No	No	Yes	Trust-anchor; Audit-log; Attribute-registry; Policy-store; Coordination
Zhang et al. [107] (2024)	IoT	Yes	No	Yes	Yes	Policy-eval; Policy-store; Trust-anchor; Audit-log; Governance
Chen et al. [108] (2025)	IoT	Yes	No	Partial	No	–
Fang et al. [109] (2025)	IoT	Yes	Yes	Yes	Yes	Policy-eval; Trust-anchor; Audit-log; Governance
Fedrecheski et al. [110] (2025)	IoT	Yes	Yes	Partial	Yes	Trust-anchor; Attribute-registry
Kavitha and Gayathri Devi [111] (2025)	IoT	Partial	Yes	Yes	Yes	Policy-eval; Trust-anchor; Audit-log; Attribute-registry; Policy-store; Governance; Coordination
Ravalika et al. [112] (2025)	IoT	Yes	No	Yes	Yes	Trust-anchor; Policy-store; Governance; Coordination; Audit-log
Stodt et al. [113] (2025)	IoT	Yes	Yes	Yes	Yes	Policy-store; Trust-anchor; Audit-log; Governance; Coordination
Waheed et al. [114] (2025)	IoT	No	Yes	Yes	Yes	Policy-eval; Trust-anchor; Audit-log; Attribute-registry; Policy-store
Akram et al. [115] (2026)	IoT	Yes	No	Yes	No	–
Díaz-Sánchez et al. [116] (2026)	IoT	Yes	Yes	Yes	No	–
Goswami and Kadao [117] (2026)	IoT	No	Partial	Partial	Yes	Policy-eval; Policy-store; Trust-anchor; Audit-log
Han et al. [118] (2026)	IoT	Yes	No	Yes	Yes	Policy-store; Trust-anchor; Audit-log; Governance
Kang et al. [119] (2026)	IoT	No	Yes	Yes	Yes	Policy-eval; Policy-store; Trust-anchor; Audit-log; Coordination
Kumar et al. [120] (2026)	IoT	No	No	Partial	Yes	Policy-eval; Trust-anchor; Audit-log; Coordination
Mailapalli and Sheela [121] (2026)	IoT	No	Yes	Yes	Yes	Policy-eval; Policy-store; Trust-anchor; Audit-log; Governance
Upadhyay et al. [122] (2026)	IoT	No	Yes	Yes	Yes	Policy-eval; Policy-store; Trust-anchor; Audit-log; Governance
Zhang et al. [123] (2026)	IoT	No	Yes	Yes	Yes	Policy-eval; Policy-store; Attribute-registry; Audit-log; Trust-anchor
Kim et al. [124] (2020)	Edge	Yes	No	Yes	No	–
Algarni et al. [125] (2021)	Edge	No	Yes	Yes	Yes	Policy-store; Trust-anchor; Audit-log; Coordination
Dougherty et al. [126] (2021)	Edge	Yes	No	Yes	No	–
Zhu et al. [127] (2021)	Edge	No	No	Yes	Yes	Policy-eval; Trust-anchor; Audit-log; Attribute-registry; Policy-store; Governance; Coordination
Frimpong et al. [128] (2022)	Edge	Partial	Yes	Yes	No	–
Zhu et al. [129] (2022)	Edge	No	Yes	Yes	Yes	Policy-eval; Trust-anchor; Audit-log; Attribute-registry; Policy-store; Governance; Coordination
Alzahrani et al. [130] (2023)	Edge	Yes	No	Yes	No	–
Pathak et al. [131] (2023)	Edge	Yes	Yes	Yes	Yes	Policy-eval; Policy-store; Trust-anchor; Audit-log
Song et al. [132] (2023)	Edge	Yes	No	Partial	No	–
Zhou et al. [133] (2023)	Edge	No	No	Yes	No	–
Andalib et al. [134] (2024)	Edge	No	Yes	Partial	Yes	Policy-eval; Policy-store; Trust-anchor; Audit-log
Liu et al. [135] (2024)	Edge	Yes	No	Yes	No	–
Ellouze et al. [136] (2025)	Edge	No	No	Partial	Yes	Policy-eval; Policy-store; Attribute-registry; Trust-anchor; Audit-log; Coordination
Sheetal et al. [137] (2025)	Edge	No	Yes	Partial	Yes	Policy-eval; Trust-anchor; Audit-log; Attribute-registry; Policy-store; Coordination
Chen et al. [138] (2026)	Edge	Yes	No	Partial	No	–
Ullah et al. [139] (2026)	Edge	Yes	Yes	Yes	Yes	Trust-anchor; Audit-log; Coordination
Shafagh et al. [140] (2020)	WSN	Yes	No	Yes	Yes	Policy-store; Trust-anchor; Audit-log
Trabelsi et al. [141] (2023)	WSN	No	No	Partial	Yes	Policy-eval; Policy-store; Attribute-registry; Trust-anchor; Audit-log; Coordination
dos Santos et al. [142] (2026)	WSN	No	Yes	Yes	Yes	Policy-eval; Trust-anchor; Audit-log; Policy-store; Governance
Jyosthna et al. [143] (2026)	WSN	No	No	Yes	No	–
Kchaou et al. [144] (2020)	V2X	Yes	Partial	Yes	Yes	Policy-store; Audit-log; Governance; Trust-anchor
Lu et al. [145] (2026)	V2X	No	Yes	Partial	No	–
Zhi et al. [146] (2026)	V2X	Yes	Yes	Yes	No	–

**Table 13:** Overall distribution of the C1–C6 classification results across the 90 selected studies.

ID	Criterion	Distribution, *n* (%)
C1	Policy Governance	Owner 40 (44.4%); Admin 32 (35.6%); Hybrid 8 (8.9%); Delegated 6 (6.7%); Consortium 4 (4.4%)
C2	Decision Basis	ABAC 42 (46.7%); Rule-based 37 (41.1%); RBAC 10 (11.1%); ReBAC 1 (1.1%)
C3	Identity and Attribute Management	Distributed 46 (51.1%); Centralized 17 (18.9%); Federated 16 (17.8%); User-held 11 (12.2%)
C4	PDP–PEP Architecture	Distributed PDP/Local PEP 58 (64.4%); Central PDP/Local PEP 12 (13.3%); Hierarchical PDP/Local PEP 10 (11.1%); On-resource decision + enforcement 10 (11.1%)
C5	Trust Establishment	Ledger-anchored integrity 45 (50.0%); Consortium trust 16 (17.8%); Local administrative trust 15 (16.7%); SSI (DID/VC) 6 (6.7%); Federation trust 5 (5.6%); PKI-based trust 3 (3.3%)
C6	Operational Assurance and Auditability	Tamper-evident log 67 (74.4%); Policy change control 55 (61.1%); Verifiable audit 26 (28.9%); Central logging 1 (1.1%); none reported 12 (13.3%)

Note: C1–C5 use mutually exclusive primary labels. C6 is multi-label; therefore, C6 counts and percentages are not mutually exclusive and do not sum to 100%.

**Table 14:** Sensitivity analysis of C2 decision-basis frequencies when CAP-BAC is treated as a separate model class.

C2 Category	Primary Coding, n (%)	CAP-BAC as Separate Class, n (%)
ABAC	42 (46.7)	35 (38.9)
Rule-based	37 (41.1)	17 (18.9)
RBAC	10 (11.1)	10 (11.1)
ReBAC	1 (1.1)	1 (1.1)
CAP-BAC	–	27 (30.0)

Note: In the sensitivity analysis, all studies coded with the CAP-BAC C4 subtype are reassigned to a separate CAP-BAC C2 category; all other C2 classifications remain unchanged.

**Table 15:** Distribution of correspondence with the authorization-relevant aspects of the selected ZTA tenets.

Tenet	Yes	Partial	No
T3: Session-bounded access	48 (53.3%)	4 (4.4%)	38 (42.2%)
T4: Dynamic policy inputs	46 (51.1%)	6 (6.7%)	38 (42.2%)
T6: Dynamic authorization enforcement	63 (70.0%)	25 (27.8%)	2 (2.2%)

**Table 16:** Correspondence between C4 architectural placement and the selected ZTA tenets.

C4 Architectural Pattern	*n*	T3 Yes, *n* (%)	T4 Yes, *n* (%)	T6 Yes, *n* (%)
Central PDP/Local PEP	12	9 (75.0%)	3 (25.0%)	9 (75.0%)
Distributed PDP/Local PEP	58	28 (48.3%)	31 (53.4%)	43 (74.1%)
Hierarchical PDP/Local PEP	10	5 (50.0%)	10 (100.0%)	7 (70.0%)
On-resource decision + enforcement	10	6 (60.0%)	2 (20.0%)	4 (40.0%)

Note: Percentages are normalized within each C4 architectural pattern and report the proportion coded as Yes. Partial and No classifications remain reported in Table 12.

## Data Availability

The data supporting the findings of this study are available within the article, its Appendix A, Appendix B and Appendix C, and the Supplementary Materials.

## Supplementary Materials

The following supporting information can be downloaded at https://www.mdpi.com/article/10.3390/s26206522/s1, one supplementary Excel workbook containing Table S1 (study-level coding matrix), Table S2 (inter-coder reliability analysis and the 20-study reliability subset), Table S3 (worked coding examples and coding guidance), and Table S4 (sensitivity analyses).

## Abbreviations

The following abbreviations are used in this manuscript:

ABAC	Attribute-Based Access Control
ACL	Access Control List
CAP-BAC	Capability-Based Access Control
CDM	Continuous Diagnostics and Mitigation
DAC	Discretionary Access Control
DID	Decentralized Identifier
DLT	Distributed Ledger Technology
DTE	Domain and Type Enforcement
IDM	Identity Management
IoT	Internet of Things
JWT	JSON Web Token
LDAP	Lightweight Directory Access Protocol
LOMAC	Low-Water-Mark Mandatory Access Control
MAC	Mandatory Access Control
NGAC	Next Generation Access Control
OIDC	OpenID Connect
OPA	Open Policy Agent
PA	Policy Administrator
PAP	Policy Administration Point
PDP	Policy Decision Point
PE	Policy Engine
PEP	Policy Enforcement Point
PIP	Policy Information Point
PKI	Public Key Infrastructure
RBAC	Role-Based Access Control
ReBAC	Relationship-Based Access Control
SC	Smart Contract
SIEM	Security Information and Event Management
SMS	Systematic Mapping Study
SSI	Self-Sovereign Identity
UCON	Usage Control
V2X	Vehicle-to-Everything
VC	Verifiable Credential
WSN	Wireless Sensor Network
XACML	eXtensible Access Control Markup Language
ZTA	Zero Trust Architecture

## Appendix A. Platform-Specific Search Queries

This appendix reports the platform-specific search queries used to construct the final mapping corpus. The five search sources shared the broad conceptual focus of distributed or decentralized authorization and access control, but the exact query formulations were not lexically identical across platforms. The main search process used Google Scholar, IEEE Xplore, the ACM Digital Library, Springer Nature Link, and Scopus. To ensure a common publication window across all sources, eligible publications were restricted to those published from 2020 through 30 June 2026. Records first published after this cutoff were excluded from the study-selection process when later searches were conducted. A separate ReBAC-oriented sensitivity search was also conducted to assess potential retrieval bias and is reported in Appendix A.6.

### Appendix A.1. Google Scholar

**Search date:** 24 August 2026
**Publication period:** 2020–2026 (eligible publications through 30 June 2026)
**Sort:** Relevance
**Retrieval:** First 10 relevance-ranked result pages
**Query:**

"distributed authorization"
OR "decentralized authorization"
OR "distributed access control"
OR "decentralized access control"

**Records retrieved:** 196
Five records published outside the predefined period were removed, leaving 191 Google Scholar records entering the study-selection process.

### Appendix A.2. IEEE Xplore

**Search date:** 23 June 2026
**Search field:** All Metadata
**Publication period:** 2020–2026
**Query:**

"distributed access control"
OR "decentralized access control"
OR "decentralized authorization"

**Records retrieved:** 114

### Appendix A.3. ACM Digital Library

**Search date:** 23 June 2026
**Publication period:** 2020–2026
**Query:**

("decentralized authorization" OR "distributed authorization")
AND ("access control")

**Records retrieved:** 46
One record published outside the predefined period was removed, leaving 45 ACM Digital Library records entering the study-selection process.

### Appendix A.4. Springer Nature Link

**Search date:** 22 June 2026
**Publication period:** 2020–2026 (eligible publications through 30 June 2026)
**Query:**

"distributed access control" AND "authorization"

**Records retrieved:** 128

### Appendix A.5. Scopus

**Search date:** 1 September 2026
**Search field:** TITLE-ABS-KEY
**Publication period:** 2020–2026 (eligible publications through 30 June 2026)
**Query:**

TITLE-ABS-KEY(
"distributed authorization"
OR "decentralized authorization"
OR "distributed access control"
OR "decentralized access control"
)

**Records retrieved:** 366

Five retrieved records fell outside the predefined publication period and were excluded, leaving 361 Scopus records entering the study-selection process.

### Appendix A.6. ReBAC-Oriented Sensitivity Search

The ReBAC-oriented sensitivity searches used platform-specific syntax adaptations where necessary. To avoid requiring studies to explicitly use distributed/decentralized authorization terminology, these terms were intentionally omitted from the sensitivity-search query. Instead, ReBAC-specific terms were combined with the predefined application-domain terms so that potentially relevant ReBAC studies could be retrieved even when they did not describe themselves explicitly as distributed authorization studies.

The Springer Nature Link search retrieved 29 records, among which two genuine ReBAC studies were identified. Both had already been identified before the sensitivity search and were subsequently excluded because their primary application domains fell outside the predefined scope. The IEEE Xplore search retrieved one ReBAC study, which did not satisfy the distributed-authorization eligibility requirements. The ACM Digital Library search retrieved 21 records. Five were proceedings-level records rather than individual research studies, leaving 16 studies for title/abstract screening. One study proceeded to full-text assessment but was excluded because a qualifying distributed authorization mechanism or function could not be established.

Accordingly, the ReBAC-oriented sensitivity searches identified no additional eligible ReBAC study for the final mapping corpus. The sensitivity-search retrieval counts were not added to the main-search totals reported in Figure 3, because these searches were conducted as targeted terminology-bias checks rather than as additional independent search sources.

## Appendix B. Inclusion and Exclusion Criteria

This appendix summarizes the inclusion and exclusion criteria applied during data cleaning, title and abstract screening, full-text retrieval, and full-text eligibility assessment. The criteria were used to identify original research studies that describe qualifying distributed authorization models, mechanisms, or architectures within the predefined application-domain scope and provide sufficient information for systematic mapping under criteria C1–C6 and the selected ZTA indicators T3, T4, and T6.

**Inclusion criteria:**

- IC1: The study proposes, implements, or evaluates an authorization model, mechanism, or architecture.
- IC2: The authorization design operates across distributed components, administrative domains, authorities, or enforcement locations.
- IC3: Authorization constitutes a primary or structurally significant contribution of the study rather than an incidental supporting function.
- IC4: The study provides sufficient information to identify PDP–PEP placement or functionally equivalent authorization decision and enforcement components.
- IC5: The study provides sufficient information to assign the applicable C1–C6 labels and to code correspondence with the selected ZTA indicators according to the predefined rules.
- IC6: The primary application area falls within the application-domain scope of IoT, edge/fog computing, sensor/WSN environments, or vehicular/V2X systems.
- IC7: The study is an original research publication or eligible research preprint first made publicly available from 2020 through 30 June 2026.

**Exclusion criteria:**

- EC1: The study primarily addresses authentication, privacy, secure communication, or data sharing, while authorization is not a central contribution.
- EC2: Authorization is mentioned only as a supporting mechanism and is not described in sufficient structural or semantic detail.
- EC3: DLT or blockchain is used only for general-purpose storage, logging, or data integrity without an authorization-relevant design.
- EC4: The study does not distribute any authorization-relevant function across multiple components or administrative domains and therefore falls outside the defined scope of distributed authorization.
- EC5: The study lacks sufficient detail on authorization policies, decision logic, identity or attribute handling, trust assumptions, or architectural components to support coding under the predefined criteria.
- EC6: The primary application area falls outside IoT, edge/fog computing, sensor/WSN environments, and vehicular/V2X systems.
- EC7: The publication is non-original or secondary literature, including a review or survey, white paper, technical report, thesis or dissertation, book, or non-research book chapter. Original conference papers published in proceedings series such as LNCS, LNNS, and CCIS are treated as conference papers and are not excluded solely because bibliographic databases represent them as book chapters.
- EC8: The record is a duplicate or describes substantially the same authorization model as a more recent or more complete publication retained in the mapping corpus.
- EC9: The full text could not be obtained through the available institutional-access or open-access sources.
- EC10: The full text is not available in English.

Records classified under EC9 passed title and abstract screening but could not proceed to full-text eligibility assessment. Accordingly, they are reported in Figure 3 as reports not retrieved rather than as reports excluded during full-text assessment. In contrast, records classified under EC10 were retrieved and assessed, and are therefore counted among the full-text exclusions.

## Appendix C. Coding Rules

### Appendix C.1. General Coding Rules

- **Predefined labels.** Each criterion is coded using its predefined label set. New labels are not introduced during study-level coding.
- **Primary labels and subtypes.** C1–C3 and C5 are assigned one primary label according to the applicable coding rules. C4 is assigned exactly one primary architectural placement pattern and zero or more non-mutually exclusive subtypes. C6 may contain more than one label when multiple operational-assurance or auditability mechanisms are explicitly reported.
- **Tie-breaking.** When more than one primary label appears plausible, the assigned label reflects the mechanism presented as primary in the study’s architecture, workflow, or design description. Secondary characteristics are recorded in the coding notes or in applicable multi-label fields.
- **Evidence-based coding.** Coding decisions are based on mechanisms explicitly reported in the full text of the corresponding studies. Evidence locations, such as sections, pages, tables, or figures, are recorded where applicable. When a design characteristic is ambiguous or insufficiently specified, the more conservative applicable label is assigned rather than inferring unreported functionality.
- **Treatment of unreported, inapplicable, and insufficiently specified information.** The coding distinguishes between a mechanism that is not reported, a field that is not applicable, and evidence that is insufficient to support a stronger classification. For the primary dimensions C1–C5, each included study is assigned an applicable primary label; studies lacking sufficient information for reliable classification were excluded during full-text eligibility assessment rather than assigned an “Undetermined” label. For C6, **– (Not specified)** indicates that no applicable operational-assurance or auditability mechanism is explicitly reported and does not imply that such a mechanism is absent from the implemented system. For conditional supplementary fields, such as DLT/SC functional roles, no role tag is assigned when the corresponding technology is not used. For the selected ZTA indicators, **No** and **Partial** are assigned according to the indicator-specific evidence rules below and should not be interpreted as proof that an unreported mechanism is absent from the implemented system.
- **Logical rather than physical placement.** Architectural placement is coded according to the logical authorization decision and enforcement functions and their relationship to the protected resource. In particular, PEP placement is determined by the component that logically controls access to the protected resource, rather than by the physical number of nodes involved in cryptographic processing, coordination, communication, or verification.
- **Primary application area.** When a study addresses multiple application areas, the descriptive area label is assigned according to the deployment context emphasized as the study’s principal target. Application-domain eligibility is assessed separately under IC6 and EC6; the descriptive area label itself does not alter the C1–C6 coding. Secondary application contexts are recorded in the coding notes where applicable.
- **Primary exclusion reason.** When a record satisfies more than one exclusion criterion, one primary exclusion reason is assigned according to the criterion that most directly determines exclusion. This prevents double counting in the study-selection flow while allowing additional observations to be retained in the screening or coding notes.

### Appendix C.2. Decision Rules for Borderline and Hybrid Cases

To improve coding transparency and reduce ambiguity, we define the following decision rules for commonly encountered borderline or hybrid cases.

Rule 1: Token usage does not imply CAP-BAC

The mere use of tokens, such as OAuth tokens, JSON Web Tokens (JWTs), or bearer tokens, does not automatically qualify a model as Capability-Based Access Control (CAP-BAC). A model is coded as CAP-BAC only when an authorization grant represents the permitted operation or resource access and can be independently validated by the enforcement side without requiring a new issuer-side authorization decision for each request. If tokens are used while authorization decisions remain issuer- or PDP-centric, the model is classified according to its decision basis (C2) and placement pattern (C4), while token use is recorded only as a C4 subtype.

Rule 2: On-chain evaluation does not redefine C4 placement

When policy evaluation logic is deployed on a distributed ledger or smart-contract platform (an “on-chain PDP”), but enforcement occurs at a local resource-side PEP, the model is classified according to its primary placement pattern, typically **Distributed PDP/Local PEP**. Ledger-based storage, replicated execution, or consensus-based policy evaluation does not by itself alter PEP placement. Ledger-related mechanisms are recorded using C4 subtypes and DLT/SC usage tags.

Rule 3: Governance (C1) is determined by effective authority

If a system is described as distributed or decentralized but policy creation, modification, and revocation remain controlled by a single administrative entity, it is coded as **Admin** under C1. **Owner** is used when resource owners retain effective policy authority over their own resources, **Consortium** when policy authority is structurally shared among participating organizations, and **Delegated** when policy authority is formally transferred to another entity. **Hybrid** is used when the reported governance structure combines multiple authority patterns without one clearly dominating. C1 therefore reflects effective control over the policy lifecycle rather than the distribution of technical components.

Rule 4: Architectural distribution does not determine trust establishment

C4 and C5 are coded independently. A system with distributed decision components may still rely on a centralized certificate authority, identity provider, consortium membership service, or other common trust root. Conversely, ledger-anchored, SSI-based, federated, or consortium trust mechanisms may be used with different C4 placement patterns. Accordingly, distributed PDP placement does not by itself determine the C5 classification.

### Appendix C.3. C1: Policy Governance

Primary label set (single-label)

- **Admin:** A centralized administrator, platform operator, or organizational authority primarily defines, modifies, or revokes authorization policies.
- **Owner:** A resource, device, service, or data owner primarily defines, modifies, or revokes policies for assets under its control.
- **Consortium:** Policy authority is jointly exercised by multiple organizations or participating authorities through an explicitly shared governance arrangement, such as voting, threshold approval, or consortium-defined rules.
- **Hybrid:** Policy governance combines two or more active loci of authority, such as owner-level discretion together with administrator- or consortium-level control, without one authority pattern clearly dominating.
- **Delegated:** Authority to define, modify, revoke, or further delegate authorization policy or authorization rights is explicitly transferred to another entity.

Primary and secondary governance labels

Each study is assigned one C1 primary label representing the governance arrangement with primary effective authority over authorization-policy creation, modification, and revocation. When a materially distinct additional governance mechanism explicitly participates in the authorization process, one optional C1 secondary label may also be assigned using the same controlled vocabulary: Admin, Owner, Consortium, Hybrid, or Delegated. The primary label is used for the main corpus-level frequency analysis, while the secondary label is retained for study-level reporting and analysis of hybrid governance arrangements. The secondary field is left blank when no materially distinct second governance mechanism is identified.

Coding rule

Delegated is assigned only when delegation of policy or authorization authority is a defining governance mechanism and the delegated entity can exercise that authority within the stated scope. The mere transfer or presentation of an access token, capability, or credential does not by itself constitute delegated governance. When multiple forms of authority jointly govern policy and no single form clearly dominates, code Hybrid unless the study explicitly defines a consortium-governance structure. A secondary label is assigned only when the additional governance mechanism materially participates in policy authority; the mere presence of another actor or administrative component is insufficient.

Relation to MAC and DAC

MAC and DAC primarily distinguish how access permissions are controlled within an access control model. They do not fully represent the allocation of policy authority across administrators, owners, consortium members, and delegated entities in distributed or multi-domain environments. Accordingly, C1 uses the governance labels above as its primary coding scheme, while MAC- or DAC-like characteristics may be recorded in the coding notes where relevant.

### Appendix C.4. C2: Decision Basis

Primary label set (single-label)

- **RBAC:** Authorization decisions are primarily determined by roles, role hierarchies, or role–permission assignments.
- **ABAC:** Authorization decisions are primarily determined by attributes of subjects, resources, actions, or the environment and by predicates evaluated over those attributes.
- **ReBAC:** Authorization decisions are primarily determined by explicitly modeled relationships or relationship paths among entities.
- **Rule-based:** Authorization decisions are expressed through rules, conditions, or other decision logic but are not clearly instantiated as RBAC, ABAC, or ReBAC.

Primary and secondary decision bases

Each study is assigned one C2 primary label corresponding to the mechanism that principally determines the authorization outcome. When a materially distinct second decision basis explicitly participates in the authorization process, one optional C2 secondary label may also be assigned using the same controlled vocabulary: RBAC, ABAC, ReBAC, or Rule-based. The primary label is used for the main corpus-level frequency analysis, while the secondary label is retained for study-level reporting and analysis of hybrid decision mechanisms. When no materially distinct secondary decision basis is identified, the secondary field is left blank.

Rule-based subtypes

When the primary C2 label is **Rule-based**, one subtype is assigned to characterize the principal rule semantics:

- **Policy-rule:** Authorization is evaluated through explicit policy, ACL, or rule-engine logic that is not clearly instantiated as RBAC, ABAC, or ReBAC.
- **Grant-rule:** Permission is principally determined by rules associated with tokens, capabilities, grants, or authorization state.
- **Context-rule:** Permission is principally determined by changing contextual conditions such as time, location, device or application state, trust or risk, or environmental state.
- **Cryptographic-condition:** Access is principally determined by cryptographic eligibility, decryptability, proof, or equivalent cryptographic conditions.
- **Other:** Used only when none of the above subtypes adequately describes the reported rule semantics; the rationale is documented in the coding notes.

Coding rule

A study is coded as **ReBAC** only when explicitly modeled relationships or relationship paths are evaluated as part of the authorization decision. Ownership, delegation, or other relationships do not by themselves constitute ReBAC unless the relationship itself forms part of the decision semantics. A rule-based implementation is coded as **ABAC** when authorization is principally determined by predicates over explicitly defined attributes, even when those predicates are implemented as rules. When the decision logic is not clearly instantiated as RBAC, ABAC, or ReBAC, it is coded as **Rule-based**. If multiple decision mechanisms are present, the mechanism that principally determines the authorization outcome is assigned as the primary label, while a materially distinct additional mechanism may be recorded as the secondary label.

### Appendix C.5. C3: Identity and Attribute Management

Primary label set (single-label)

- **Centralized:** A single authority primarily issues, maintains, or provides the identity and attribute information required for authorization.
- **Federated:** Multiple independently managed domains, identity providers, issuers, or attribute authorities provide identity and attribute information through explicit cross-domain trust relationships.
- **Distributed:** Identity- or attribute-management functions or authoritative state are allocated across multiple authorities, registries, or replicated infrastructures rather than being controlled by a single primary management authority.
- **User-held:** Users or subjects possess authorization-relevant credentials or attributes and present them, or corresponding cryptographic proofs, to verifiers as the primary means of supplying authorization information.

Primary and secondary identity/attribute-management labels

Each study is assigned one C3 primary label representing the principal arrangement through which authorization-relevant identity and attribute information is issued, maintained, obtained, or presented. When a materially distinct second arrangement explicitly participates in the authorization process, one optional C3 secondary label may also be assigned using the same controlled vocabulary: Centralized, Federated, Distributed, or User-held. The primary label is used for the main corpus-level frequency analysis, while the secondary label is retained for study-level reporting and analysis of hybrid identity- and attribute-management arrangements. The secondary field is left blank when no materially distinct second arrangement is identified.

Coding rule

**Federated** is used when independently managed domains exchange or accept identity and attribute information through established federation relationships. **Distributed** is used when management functions or authoritative state are distributed across multiple authorities, registries, or replicated components without a single primary management authority. **User-held** is used when holder-mediated possession and presentation of credentials or proofs is a defining feature of the authorization process, even though credential issuance, revocation, or status information may still depend on external authorities. When multiple arrangements are present, the arrangement that principally supplies authorization-relevant identity or attribute information is assigned as the primary label, while a materially distinct additional arrangement may be recorded as the secondary label.

### Appendix C.6. C4: PDP–PEP Architecture

Primary placement patterns (single-label)

- **Central PDP/Local PEP (C4-a):** A logically centralized PDP makes the authorization decision, while a PEP associated with the protected service or resource enforces that decision. Other functions, such as identity management, trust establishment, credential issuance, or audit logging, may still be distributed.
- **Distributed PDP/Local PEP (C4-b):** Multiple logically distinct decision components, replicated decision functions, or independently deployed PDPs participate in determining authorization outcomes across components or domains, while enforcement remains local to the protected service or resource. Distribution may involve replicated policy evaluation, coordination among decision components, or aggregation of partial authorization decisions.
- **Hierarchical PDP/Local PEP (C4-c):** Authorization decision functions are organized across multiple architectural levels. Upper-level components provide global policies, constraints, coordination, or cross-domain decisions, while lower-level components perform local decision making or validation subject to those inputs. Enforcement remains associated with the protected service or resource.
- **On-resource decision + enforcement (C4-d):** The substantive authorization decision and enforcement functions are co-located at the protected resource or within the component that directly controls access to it. The resource-side component itself evaluates the authorization conditions that determine Grant/Deny, for example, through embedded policy evaluation, evaluation of authorization conditions represented in capabilities or credentials, or cryptographic eligibility checks.

Primary-pattern coding rule

Each study is assigned exactly one primary C4 placement pattern according to the logical placement and interaction of authorization decision and enforcement functions. C4 does not use a secondary placement label; additional architectural characteristics are represented separately using non-mutually exclusive C4 subtypes. The physical number of nodes, replicas, validators, or blockchain participants does not by itself determine the primary C4 pattern. When distinct upper- and lower-level authorization decision stages are present, the architecture is coded as **Hierarchical PDP/Local PEP**; when multiple peer or replicated decision functions participate without such a hierarchical decision structure and enforcement remains local, it is coded as **Distributed PDP/Local PEP**. Local validation of an authorization grant already determined by an upstream PDP is treated as local enforcement and does not by itself qualify for the **On-resource decision + enforcement** pattern. This category requires the resource-side component itself to perform the substantive authorization decision as well as enforcement.

C4 subtypes (multi-label)

In addition to the primary PDP–PEP placement pattern, zero or more non-mutually exclusive C4 subtypes are assigned when the corresponding mechanism is explicitly present and materially participates in the authorization workflow.

- **Token-based:** Authorization-related tokens or credentials are used to convey access rights, scopes, grants, authorization decisions, or other authorization-relevant state between decision and enforcement components. Token use alone does not imply CAP-BAC.
- **Crypto-enforced:** Access eligibility is materially enforced through a cryptographic condition or mechanism, such as decryptability, proof verification, cryptographic key release, or another cryptographic operation that directly determines whether the protected resource or operation can be accessed. The use of signatures, hashes, or encryption solely for authentication, integrity protection, or secure communication is insufficient.
- **CAP-BAC:** A capability represents an authorization grant for a protected resource or operation and can be independently validated by the enforcement side without requiring a new issuer-side authorization decision for each request. The mere use of a bearer token, access token, credential, or identity claim does not by itself constitute CAP-BAC.
- **On-chain PDP:** A DLT or smart-contract component materially performs authorization-policy evaluation, grant/deny determination, validation of authorization conditions, or authorization-related state transitions. Storage, replication, logging, or integrity protection on a ledger alone is insufficient.
- **Aggregated-at-gateway:** A gateway, hub, edge/fog node, proxy, or equivalent intermediary aggregates authorization-related communication, decision processing, validation, or enforcement functions on behalf of multiple subordinate devices, services, or protected resources. A component is not assigned this subtype merely because it relays traffic or is physically located between a client and a resource.
- **Multi-authority:** Multiple distinct authorities, issuers, attribute authorities, policy authorities, or authorization decision entities materially participate in the authorization process. Replication of the same logical authority across multiple nodes does not by itself qualify. Multi-authority participation does not necessarily imply federation trust under C5.
- **SSI:** An SSI-oriented credential architecture, such as one using DIDs, Verifiable Credentials, Verifiable Presentations, or equivalent holder-mediated credentials, materially participates in the authorization workflow. The subtype reflects the architectural use of SSI mechanisms and does not by itself determine the C2 decision basis or C5 trust label.

Subtype coding rule

Assign all applicable C4 subtypes supported by the reported architecture or authorization workflow. The subtypes characterize additional architectural and enforcement mechanisms and do not replace or alter the single primary C4 placement pattern.

**Token-based** and **CAP-BAC** may be assigned together when a capability is implemented as a token or credential. However, Token-based is broader: CAP-BAC additionally requires an authorization grant that can be independently validated by the enforcement side. Similarly, **On-chain PDP** is assigned only when a ledger or smart contract materially participates in authorization evaluation or decision processing; ledger-based policy storage, trust anchoring, coordination, or audit logging alone does not qualify.

**Aggregated-at-gateway** requires an authorization-relevant aggregation function for multiple subordinate endpoints or resources rather than ordinary network forwarding. **Multi-authority** requires materially distinct authorization-relevant authorities rather than multiple replicas or validators of a single authority. **Crypto-enforced** requires cryptography to participate directly in access eligibility or enforcement rather than merely protecting messages, tokens, or stored data.

When evidence for a subtype is ambiguous or the mechanism is only incidental to the authorization workflow, the subtype is not assigned and the uncertainty may instead be documented in the coding notes.

### Appendix C.7. C5: Trust Establishment

Primary label set (single-label)

- **Local administrative trust:** Authorization-relevant identities, attributes, policies, or decisions are accepted within a single administrative domain based on locally managed accounts, system controls, or administrative authority, without an explicit cross-domain trust mechanism.
- **PKI-based trust:** Trust is primarily established through certificate validation and certificate-based public-key bindings under an explicit PKI or equivalent certificate-authority/root trust framework.
- **Federation trust:** Trust is established through explicit inter-domain federation relationships among identity providers, issuers, service providers, or administrative domains, such as SAML- or OIDC-based federation.
- **SSI (DID/VC):** Authorization-relevant trust is primarily established through the verification of DIDs, Verifiable Credentials, and their issuers. A ledger or registry may support DID resolution, credential metadata, or status information, but holder-presented verifiable credentials constitute the principal trust mechanism.
- **Consortium trust:** Trust is primarily established through membership in a permissioned consortium and its shared governance, admission, validation, or consensus arrangements. Participating organizations jointly determine which entities, authorities, or authorization-relevant state are accepted.
- **Ledger-anchored integrity:** A replicated ledger serves as the principal integrity anchor for authorization-relevant policies, attributes, credentials, decisions, or audit records. The label concerns reliance on ledger-maintained reference state and does not by itself imply consortium governance or decentralized policy authority.

Primary and secondary trust labels

Each study is assigned one C5 primary label representing the principal mechanism through which authorization-relevant identities, attributes, credentials, policies, decisions, or state are accepted as trustworthy. When a materially distinct second trust mechanism explicitly participates in the authorization workflow, one optional C5 secondary label may also be assigned using the same controlled vocabulary: Local administrative trust, PKI-based trust, Federation trust, SSI (DID/VC), Consortium trust, or Ledger-anchored integrity. The primary label is used for the main corpus-level frequency analysis, while the secondary label is retained for study-level reporting and analysis of hybrid trust arrangements. The secondary field is left blank when no materially distinct second trust mechanism is identified.

Coding rule

Use **PKI-based trust** when certificate validation and certificate-based public-key bindings form the principal trust basis. The use of public-key cryptography, signatures, or encryption alone does not constitute PKI-based trust in the absence of an explicit certificate-based trust framework.

Use **Federation trust** when independently managed domains explicitly establish or rely on cross-domain trust relationships for identity or attribute information. The presence of multiple authorities alone does not constitute federation trust.

Use **SSI (DID/VC)** when holder-presented verifiable credentials and DID- or issuer-based verification are central to the authorization workflow, even when conventional signatures or ledger-supported registries are also used.

Use **Consortium trust** when trust primarily derives from permissioned membership and shared governance among participating organizations.

Use **Ledger-anchored integrity** when ledger-maintained reference state provides the principal integrity basis for accepting authorization-relevant information. The mere use of a blockchain or distributed ledger for storage, communication, or auxiliary logging is insufficient.

Distinction between Consortium trust and Ledger-anchored integrity

Consortium trust and Ledger-anchored integrity are distinguished by the principal basis on which authorization-relevant information is accepted as trustworthy, rather than by the blockchain or DLT platform used. **Consortium trust** is assigned when permissioned membership, organizational admission, and shared governance among participating organizations constitute the primary trust basis. **Ledger-anchored integrity** is assigned when authorization-relevant policies, attributes, credentials, decisions, or state are primarily trusted because they are anchored in replicated ledger state, without consortium membership or shared governance serving as the principal trust basis. Accordingly, the use of a permissioned blockchain platform, including Hyperledger Fabric, does not by itself imply Consortium trust.

When multiple trust mechanisms are present, the mechanism that principally establishes trust in the authorization workflow is assigned as the primary label, while one materially distinct additional mechanism may be recorded as the secondary label. Additional characteristics, where necessary, are documented in the coding notes.

### Appendix C.8. C6: Operational Assurance and Auditability

Label set (multi-label where applicable)

- **Tamper-evident log:** Authorization-related events or state changes are recorded using mechanisms intended to make unauthorized modification detectable, such as append-oriented, cryptographically linked, or ledger-backed records.
- **Verifiable audit:** The study explicitly provides mechanisms through which audit records, authorization events, or accountability claims can be independently checked, reconstructed, or verified using proofs, verifiable traces, or equivalent evidence.
- **Policy change control:** The model explicitly governs or controls changes to authorization policies or rules, including their creation, modification, deletion, approval, versioning, or controlled propagation, as well as equivalent administratively managed changes to role or permission structures that function as authorization policy.
- **Central logging:** Authorization-related events are recorded through a centralized logging mechanism, but tamper evidence or independent audit verification is not explicitly provided for that logging mechanism.
- **– (Not specified):** No applicable authorization-related operational-assurance or auditability mechanism is explicitly reported.

Coding rule

C6 is multi-label: assign all applicable labels when multiple distinct mechanisms are explicitly described in the full text. For example, a study may combine a tamper-evident log with policy change control or verifiable audit.

**Tamper-evident log** is assigned when unauthorized modification of recorded authorization events or state is intended to be detectable. The mere use of a blockchain or distributed ledger does not automatically qualify; the ledger must explicitly record or protect authorization-relevant events or state in a manner that provides tamper evidence.

**Verifiable audit** requires an additional mechanism through which the relevant authorization events, records, or accountability claims can be independently checked, proven, or reconstructed. Tamper evidence alone is therefore insufficient for this label.

**Policy change control** is assigned when the study explicitly describes controlled changes to authorization policies or rules, or equivalent administratively managed changes to role or permission structures that function as authorization policy. Qualifying changes may include creation, modification, deletion, approval, versioning, or controlled propagation. Expiration, revocation, or status checking of an already issued credential, token, capability, or individual authorization grant alone does not qualify; such lifecycle handling is evaluated under T6.

**Central logging** is assigned when authorization-related events are explicitly recorded by a centralized logging mechanism without an explicitly reported tamper-evidence or independent-verification property for that mechanism.

Assign **– (Not specified)** only when none of the other C6 labels is supported by the reported evidence. It is mutually exclusive with the other C6 labels. This designation indicates absence of explicitly reported information rather than evidence that no such mechanism exists in the implemented system.

### Appendix C.9. Selected ZTA Tenets (T3, T4, and T6)

Correspondence with the authorization-relevant aspects of T3, T4, and T6 is coded using a three-level scale: **Yes**, **Partial**, and **No**. These labels represent correspondence with the coding definitions below and do not indicate overall ZTA compliance.

T3 (Session-bounded access)

**Yes** is assigned when authorization, a session, a credential, or other authorization-relevant evidence is explicitly bounded by an operationally specified validity constraint, such as an expiration time, validity interval, lease or session duration, deadline, or limited-use/NTU condition. Renewal, refresh, or reauthorization may be described but is not required for a Yes classification.

**Partial** is assigned when bounded validity is suggested but the scope or operational meaning of the bound is not specified clearly enough to establish a concrete authorization-validity constraint, for example, when a study refers only generally to temporary or limited-period access or to expired permissions without defining the lifetime of the authorization grant, session, credential, or authorization-relevant evidence.

**No** is assigned when no explicit bounded-validity mechanism is described. Isolated request-time authorization, static permissions, or timestamps, nonces, and replay-protection mechanisms alone do not qualify as T3.

Representative T3 coding examples

The following examples illustrate the operational boundary among the three T3 classifications.

- **Yes:** Kim et al. [124] explicitly define temporary session keys, communication-session duration, and expiring migration certificates or tokens (Sec. 3.1.1–3.1.3 and Sec. 3.2.1–3.2.2). Ren et al. [64] define token validity using the number of times used (NTU), version timestamps, and access duration, with reauthorization when the NTU is exhausted (Sec. IV-A and Sec. V-D–E).
- **Partial:** Kavitha and Gayathri Devi [111] describe temporary or limited-period access, but the validity semantics of the authorization evidence are not specified precisely enough for a Yes classification (Sec. 3.9). Yan et al. [97] describe expired permissions and reputation-based blocking, but do not define a general lifetime-bounded authorization token or session model (Sec. III-A–B).
- **No:** Mishra et al. [61] include a timestamp in the authorization token but do not define expiration, a validity interval, a usage bound, or a bounded authorization session (Sec. III-B–C). Yang et al. [106] do not report an authorization-lifetime or renewal mechanism (Secs. 4–6).

T4 (Dynamic policy inputs)

T4 concerns the information used to make an authorization decision. It captures whether a dynamically changing current value is incorporated into the Grant/Deny decision.

**Yes** is assigned when one or more dynamically changing current values explicitly affect the authorization decision, such as current risk, location or time used as policy context, device or application state, posture, behavior, reputation, resource state, or environmental conditions.

**Partial** is assigned when dynamic or contextual information is described as relevant to authorization but the study does not specify clearly enough whether or how the changing value directly affects the Grant/Deny decision.

**No** is assigned when authorization decisions rely only on static roles, attributes, relationships, or rules without an explicitly changing current input to the decision. Expiration or validity checking alone is treated under T3, while mutable policy, role, revocation, or authorization state alone is treated under T6 unless it is explicitly used as a dynamic contextual input to Grant/Deny.

T6 (Dynamic authorization enforcement and lifecycle control)

T6 concerns whether access is evaluated or enforced against current authorization-relevant state and whether changes to that state can affect later access. It includes request-time or current-state validation, reauthorization, revocation or status checking, privilege changes, session invalidation, and policy- or state-triggered re-evaluation.

**Yes** is assigned when the study explicitly describes a mechanism by which current authorization-relevant state is checked or re-evaluated so that a later access attempt can be permitted or denied differently after a relevant state or lifecycle change. Examples include per-request validation against current policy, role, credential, revocation, blacklist, or authorization state; reauthorization; revocation or status propagation; privilege reduction; session invalidation; or policy-triggered re-evaluation.

**Partial** is assigned when some current-state or lifecycle handling is described, but the study does not specify clearly enough how the relevant state is propagated, checked, or enforced for later access. Examples include revocation or policy updates without a defined enforcement path, or request-time validation whose connection to mutable authorization state is only partially specified.

**No** is assigned when the study describes only an ordinary authorization check against static policy or previously issued evidence, without explicit current-state or lifecycle handling capable of changing later access. Static signature or token verification, or a fresh request evaluation alone, is insufficient unless authorization-relevant state that may have changed is explicitly checked.

Representative T6 coding examples

The following examples illustrate the operational boundary among the three T6 classifications.

- **Yes:** Kim et al. [124] require entities to repeat authorization after migration, establish trust with the new authorization service, and obtain or request new session keys (Sec. 2.3 and Sec. 3.3.2). Ren et al. [64] describe per-request token validation, reauthorization after NTU exhaustion, policy updates, explicit revocation, and rejection of obsolete or canceled tokens (Sec. V-D–F).
- **Partial:** Chen et al. [108] validate authorization evidence on each request, but attribute-revocation handling is left as future work (Sec. V-B and Sec. VI). Pateritsas and Petrakis [91] check each protected-request token through the Authorization Service before local enforcement, but do not explicitly describe how changes in authorization-relevant state, such as role or revocation status, would affect subsequent access (Sec. 3.1.5).
- **No:** Mishra et al. [61] validate the token, signature, and timestamp for a resource request but do not define revocation, reauthorization, or validation against mutable current authorization state (Sec. III-B–C). Yang et al. [106] do not report an authorization-revocation or status-validation lifecycle (Secs. 5–6).

Distinction between T4 and T6

T4 and T6 are coded independently. T4 concerns dynamically changing contextual or state information used as an input to the Grant/Deny decision, whereas T6 concerns whether access is evaluated or enforced against current authorization-relevant state so that later access can reflect authorization-state or lifecycle changes. Dynamic context at request time may therefore support T4 without supporting T6 when it is merely a decision input and no current authorization-state or lifecycle handling is described. Conversely, current policy, role, credential-status, revocation, or other authorization-state validation may support T6 without supporting T4. A mechanism may support both when changing context is explicitly re-evaluated and enforced as part of current-state authorization.

Distinction between T3 and T6

T3 and T6 capture different aspects of authorization behavior. T3 concerns whether an authorization grant, session, credential, or authorization-relevant evidence has an explicit bounded validity. T6 concerns whether access is evaluated or enforced against current authorization-relevant state so that lifecycle or state changes can affect later access. An explicit token lifetime or expiration may therefore support T3 without establishing T6 unless that validity or status is actually checked and enforced for later access. Conversely, a system may support T6 through current-state validation, reauthorization, revocation, or policy-change enforcement even when no session-bounded or expiring authorization evidence is defined.

### Appendix C.10. DLT/SC Usage Tags

Tag set (multi-label)

When DLTs and/or SCs are used, their explicitly reported authorization-related functions are coded using the following standardized tags:

- Policy-eval: DLTs or SCs materially participate in evaluating authorization conditions, validating authorization grants, or executing authorization-related state transitions.
- Trust-anchor: Ledger-maintained state anchors the integrity of authorization-relevant identities, keys, credentials, attributes, policies, or decisions.
- Audit-log: Authorization-related events or state changes are recorded using the ledger to support tamper evidence, provenance, accountability, or subsequent verification.
- Attribute-registry: DLTs or SCs maintain or reference authorization-relevant attributes, DID information, credential metadata, issuer information, or status data.
- Policy-store: Authorization policies, rules, permissions, capabilities, or related policy state are stored or replicated using the ledger.
- Governance: DLTs or SCs support membership management, voting, approval, policy administration, authority allocation, or other authorization-related governance processes.
- Coordination: DLTs or SCs coordinate authorization-related actions, evidence, state, or decisions among multiple components, authorities, or administrative domains.

Multiple tags may be assigned to a single study because the functional roles are not mutually exclusive. When a selected study does not report DLT/SC usage, no DLT/SC role tag is assigned.

Selection rule

Assign every applicable tag for which the corresponding function is explicitly performed or materially supported by a DLT or SC component. The presence of a ledger, smart contract, or blockchain platform alone is not sufficient to assign a functional tag.

No fixed maximum number of tags is imposed. When several functions are supported by the same DLT/SC infrastructure, each function is coded separately if it is materially involved in the authorization workflow and supported by the reported evidence. Ad hoc study-specific tags are not introduced during coding; unusual functions that do not fit the predefined vocabulary are documented in the coding notes.

The DLT/SC role tags are supplementary implementation descriptors and do not determine the C1–C6 labels or the selected ZTA indicators. For example, Trust-anchor records a functional use of DLT/SC, whereas C5 identifies the primary trust-establishment mechanism of the overall authorization design. Similarly, DLT/SC-based policy evaluation, state maintenance, or audit logging does not by itself establish correspondence with T6; explicit authorization-lifecycle behavior is required for the T6 coding.
